# Data-driven recommendation of agents, temperature, and equivalence ratios for organic synthesis

**DOI:** 10.1039/d5sc04957a

**Published:** 2025-09-05

**Authors:** Xiaoqi Sun, Jiannan Liu, Babak Mahjour, Klavs F. Jensen, Connor W. Coley

**Affiliations:** a Department of Chemical Engineering, Massachusetts Institute of Technology 77 Massachusetts Avenue Cambridge MA 02139 USA ccoley@mit.edu; b Department of Electrical Engineering and Computer Science, Massachusetts Institute of Technology 77 Massachusetts Avenue Cambridge MA 02139 USA

## Abstract

The identification of suitable reaction conditions is a crucial step in organic synthesis. Computer-aided synthesis planning promises to improve the efficiency of chemistry and enable robot-assisted workflows, but there remains a gap in bridging computational tools with experimental execution due to the challenge of reaction condition prediction. The conditions used to carry out a reaction consist of qualitative details, such as the discrete identities of “above-the-arrow” agents (catalysts, additives, solvents, *etc.*) as well as quantitative details, such as temperature and concentrations of both reactants (product contributing) and agents. These procedural aspects of organic chemistry exert a direct influence over the outcome of a chemical transformation and must be provided in any hypothetical autonomous synthesis workflow. In this work, we push beyond qualitative reaction condition recommendation by developing a data-driven framework that incorporates quantitative details, specifically equivalence ratios. We frame the condition recommendation problem as four sub-tasks: predicting agent identities, reaction temperature, reactant amounts, and agent amounts, and evaluate our model accordingly. We demonstrate improved performance over popularity and nearest neighbor baselines and highlight the model's practical utility for predicting conditions in diverse reaction classes *via* representative case studies.

## Abstract 2

A machine learning framework predicts suitable agents, temperature, and equivalence ratios for reactants and agents. The model consistently outperforms strong baselines, enabling more complete and automation-ready reaction protocols.

## Graphical abstract

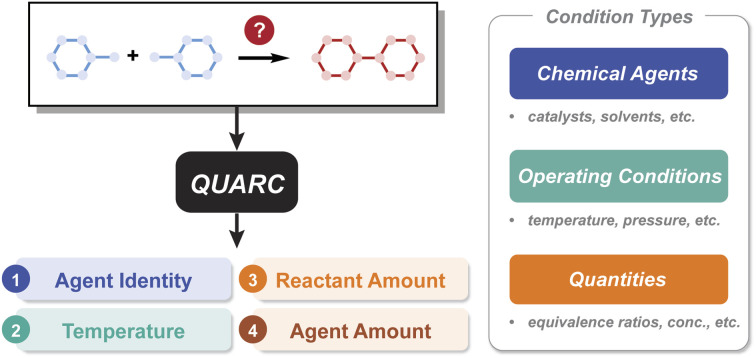

## Introduction

Small molecule synthesis drives progress in many industries such as pharmaceuticals, materials science, and agriculture as an integral part of the iterative design–make–test–analyze (DMTA) cycle used to nominate and optimize candidate compounds.^1–3^ Computer-aided synthesis planning (CASP) tools can accelerate this process by providing algorithmic, often data-driven, strategies to facilitate the design of synthetic pathways.^4–7^ Recently, CASP has seen a strong and promising resurgence in the modern age of “big data”, where computational optimization of molecular structures at the “design” stage increasingly faces a bottleneck at the “make” phase. However, while efforts in CASP have primarily focused on retrosynthetic planning to deconstruct target molecules into simpler building blocks,^6–13^ the task of predicting suitable reaction conditions to execute proposed syntheses is also of high importance.

Reaction conditions influence nearly all aspects of a reaction's outcome such as the intended product's yield, impurity distribution, and ease of purification. Here, reaction conditions are broadly defined to include chemical agents (*e.g.*, reagents, catalysts, and solvents), quantities (*e.g.*, equivalence ratios and concentrations), and operating conditions (*e.g.*, temperature, pressure, time, vessels). Incorporating reaction condition selection has been shown to improve the accuracy (and well-posedness) of product prediction models^14,15^ and can facilitate considerations of one-pot compatibility,^16,17^ green chemistry,^18,19^ and safety,^20^ all of which ultimately improve route prioritization in process chemistry.^21,22^ Further, condition prediction is essential for automated synthesis as it helps bridge the gap in specificity between retrosynthetic pathways and experimental protocols.^3,8,23–29^

In this work, we consider the goal of reaction condition recommendation to be proposing conditions that enable product formation with non-negligible yield. Alternate goals might involve identifying the “best” conditions—under various definitions—which has been the focus of substantial recent work, *e.g.*, maximizing yield,^30,31^ generality,^32,33^ or robustness.^34^ Due to data availability, these goals are not well-addressed by *a priori* model predictions, but by experimental screening and optimization campaigns. However, Shields *et al.* showed that expert-selected initializations outperform random initializations at early iterations in a Bayesian optimization campaign,^30^ suggesting that these workflows may be further improved through literature-informed initializations or design space definitions.

Beyond the context of supporting automated chemical synthesis or providing warm-starts for reaction optimization, condition recommendation models can serve as standalone tools that augment expert intuition with millions of reaction precedents found in reference databases. When retrained and deployed in the context of a pharmaceutical company, these models allow experience to be institutionalized and shared across the organization. Chemists can maintain their typical experimental screening and optimization workflows and choose to incorporate model suggestions as a source of inspiration as desired.

Current efforts in reaction condition prediction differ primarily in the assumed structure of the condition space, particularly whether condition components are assigned to predefined roles such as “catalyst” or “solvent.” We focus our discussion on the growing body of data-driven methods rather than the first-principles or physics-based methods that have been used for narrower problems such as solvent prediction.^35^ The increasing availability of standardized datasets has recently fueled the development of many machine learning approaches for reaction condition prediction. A recent review^36^ provides a complementary perspective by grouping methods according to model applicability: global models, which can in principle suggest conditions for any reaction type, and local models, which are tailored to narrower domains such as specific reaction families.

Given a fixed set of reaction roles and a predefined set of agents, condition recommendation becomes a supervised learning problem, where each role-specific slot is treated as a multiclass classification task. Machine learning models have been built to predict specific condition components for various reaction types, such as predicting solvent/catalyst,^37^ solvent-only,^38^ and phosphine ligand only.^19^

When predicting multiple condition components with fixed roles, approaches can be broadly categorized into autoregressive and non-autoregressive strategies. The autoregressive formulation generates components one by one, with later predictions conditioned on previous ones, thus capturing the interdependency among condition components. For example, Gao *et al.*^39^ defined reactions as having five agent roles (one catalyst, two reagents, and two solvents), and built a chain of classifiers. Each classifier predicts for one role and feeds the output to the next. More recently, transformer-based models^40,41^ have offered a more unified and powerful approach to this same autoregressive, role-by-role prediction. On the other hand, non-autoregressive approaches predict conditions either independently for each role or jointly for all roles at once. For the former, top candidates for each role can be enumerated and then ranked to find a complete set of conditions.^42,43^ For the latter, the task can be formulated as a multi-label classification problem over a concatenated target vector representing all roles.^44–46^ Models are trained to predict the entire set of conditions jointly in a single step. Regardless of the prediction strategy, the structured nature of a fixed-role condition space allows relatively easy integration of continuous parameters like temperature, pressure, and reaction time, though typically only temperature is included as a prediction target.

Within fixed-role paradigms, a complementary approach to condition recommendation searches a predefined, finite space of component combinations (*e.g.*, solvents, catalysts, bases). These combinations are then each scored based on predicted compatibility, either using regression models to directly predict yield^47–50^ or using ranking models to order conditions by the likelihood of success.^51^

The rigidity and potential ambiguity in assigning reaction roles have motivated approaches that relax this requirement, allowing greater flexibility in agent prediction. Classification approaches can avoid enforcing a fixed role order^52^ or a predefined number of roles by concatenating all candidate agents into a single vector. Agent prediction can also be treated as a sequence-to-sequence translation task.^12,14,53–55^ Here, models generate the SMILES strings of all chemical agents. Unlike classification approaches being restricted to a predefined list, these models use an “open” vocabulary and thus can generate potentially novel agents—arguably detracting from applications to automated synthesis. A recent direction uses Large Language Models (LLMs) to propose experimental protocols, ranging from human-readable procedures^56^ to more structured formats that are intended to be integrated with robotic platforms.^26–28,57–59^

While LLMs have shown promise in orchestrating generated conditions for synthesis planning and execution, achieving precise predictions remains a challenge. LLMs often trade off specificity for breadth. We believe that there remains a benefit of specialized condition models trained on large, curated datasets of chemical reactions that can produce precise, structured outputs. Such predictions can be readily convertible into executable instructions with heuristics, sequence-to-sequence models,^60^ or LLMs. The models themselves can also be used as tools as part of an agentic framework. However, current specialized condition models provide predictions typically only for agent identities and temperature. They fall short of capturing the richer condition information necessary for execution, such as quantities. This can be partly attributed to the scarcity of structured quantitative data in widely used reaction databases such as USPTO.^61^

**Fig. 1:**
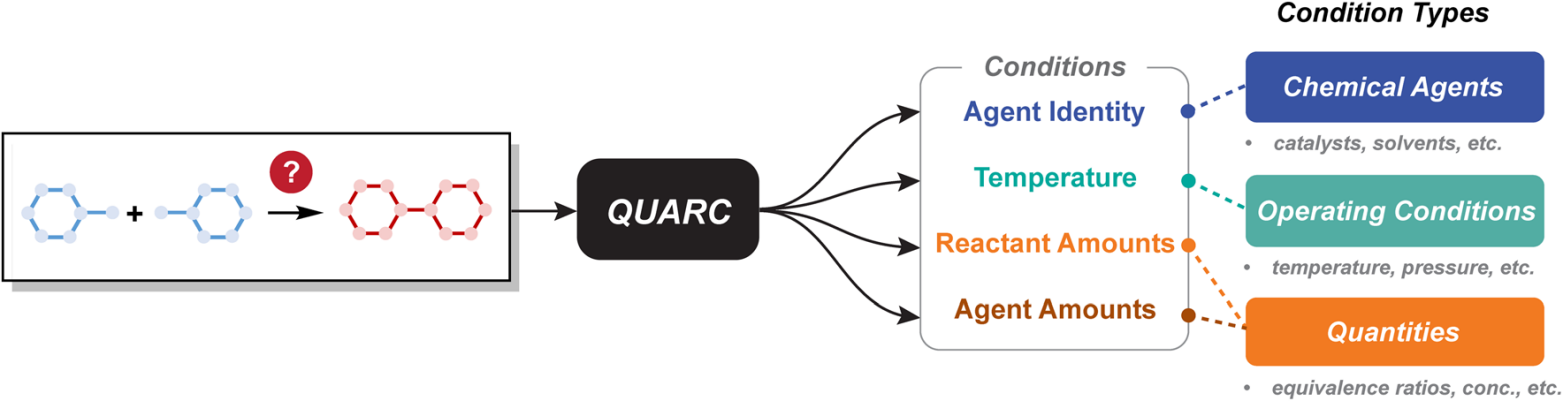
Overview of reaction condition recommendation. QUARC can suggest conditions for chemical agent identity, temperature, reactant amounts, and agent amounts, spanning the three main types of conditions: chemical agents, operating conditions, and quantities.

In this work, we introduce QUARC (QUAntitative Recommendation of reaction Conditions), a supervised model framework for quantitative reaction condition recommendation (Fig. 1). QUARC extends work that makes structured predictions, but relaxes requirements for role assignments and allows variable numbers of predictions more fluidly; equivalence ratios are also predicted by the model, ultimately yielding a structured set of reaction conditions that can easily be post-processed into code for automated execution or as the basis of a reaction optimization campaign if desired. QUARC outputs chemical agent identities, reaction temperature, and the normalized amounts of each reactant and agent, offering actionable conditions for reaction execution. We compare against chemistry-relevant baselines including popularity and nearest neighbor. While prior work has claimed that machine learning models are unable to outperform such baselines,^62^ we demonstrate that the learned models provide modest improvements across all condition prediction tasks. We further present representative examples to highlight the model's improvements over baselines.

## Methods

### Task formulation

We treat all non-reactant, non-product species uniformly as “agents” with a single vocabulary. This flexible formulation is reaction-role agnostic, thus avoiding the inherent ambiguity that arises from loosely defined roles (*e.g.*, sometimes a solvent can also be treated as a reagent).

**Fig. 2:**
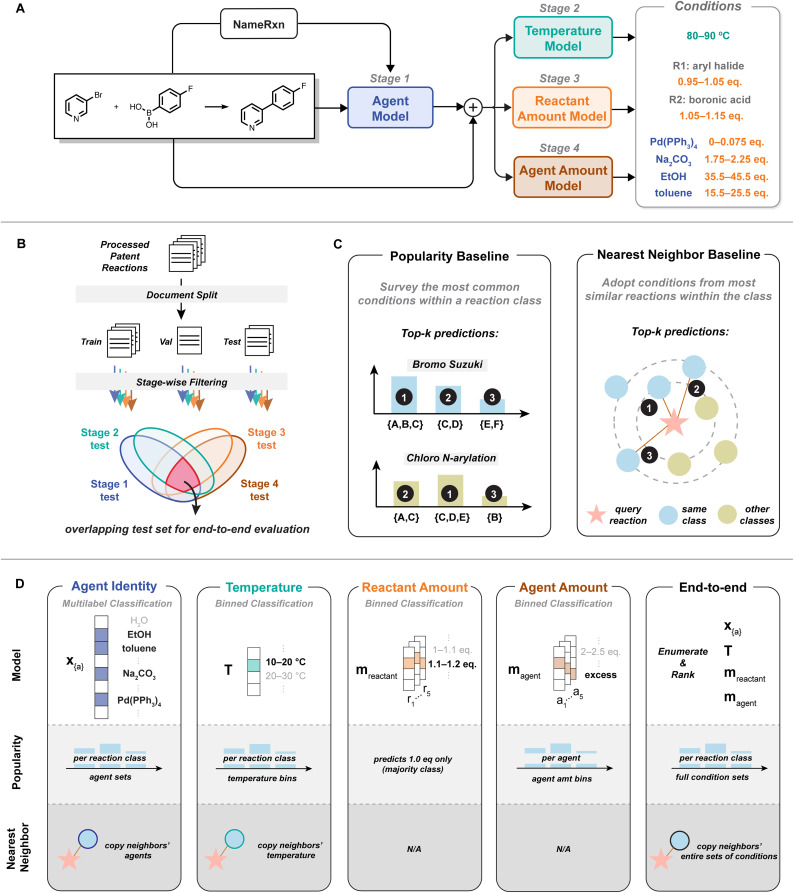
The workflow of QUARC. (A) Inference pipeline. For a query reaction, QUARC first predicts agents, then uses the predicted agents together with the reaction input to predict the temperature and the amounts for each reactant and agent. (B) Data split. Preprocessed patent reactions from Pistachio^63^ are split at the document level into training/validation/test sets, with four stage-specific test sets used to evaluate different tasks and one overlapping set for end-to-end evaluation. (C) Baselines. Popularity baseline returns the most common conditions per reaction class; nearest neighbor baseline identifies similar reactions and adopts their conditions as predictions. Reaction classes are defined using the most detailed tier of the NameRxn^64^ hierarchy. (D) Stage-wise tasks for models and baselines.

We formulate condition recommendation as a four-stage prediction task, covering agent identity, temperature, reactant amount, and agent amount (Fig. 2A). Each stage is trained independently on reactions with the appropriate subset of information as not all reaction entries have complete information. Inference is sequential: the model first predicts agents, then using the predicted agents with reactants and product(s) to determine the remaining conditions. This formulation maximizes data usage and captures natural dependencies between conditions, for example, temperature selection may depend on the presence of specific agents.

In this framework, stage 1 predicts agent identity as a multi-label classification task. A set of agents are decoded autoregressively *via* beam search during inference, producing a multi-hot vector. Stage 2 through stage 4 predict temperature, reactant amounts, and agent amounts respectively, each modeled as a binned classification task.

### Data

We use the Pistachio dataset^63^ (2023Q2 version), which contains around 15 million (4.96 million unique) reactions extracted from patents. Each entry includes reaction SMILES, component annotations (reactants, agents, products), structured quantity information (mass, moles, or volume), and a paragraph of reaction procedures. Our data preprocessing procedures include extracting key information, deduplicating reactions at the condition level, generating an agent vocabulary, splitting data at the document level into training (75%), validation (5%), and test (20%) sets, and applying stage-specific filters to curate four distinct datasets for each stage (Fig. 2B).

During data extraction, we standardize all reported quantities into moles. It is also required to resolve the quantities of species reported as mixtures or solutions. Our preprocessing pipeline handles unit conversion, solution parsing, and concentration calculation. Then we deduplicate reactions at the condition level and filter out perceived low-quality reactions based on molecular size, parsability, and component count. The agent vocabulary is defined from components that occur with measurable quantities, applying a minimum frequency threshold of 50 to exclude rare or misclassified entries. The resulting vocabulary has 1376 agents. We apply document-level split prior to stage-specific filtering to ensure the end-to-end evaluation uses held-out data. Full preprocessing details are available in Section S2. We also report the distribution of reaction types in each data split for all stages in Section S2.

### Chemistry-relevant baselines

We assess model performance against two chemistry-relevant, purely deterministic baselines (Fig. 2C).

We implement a popularity baseline^62^ that identifies the most common conditions within the specific reaction class of the query reaction. Reaction classification follows the most detailed level of the three-tier hierarchical categorization by NameRxn.^64,65^ For example, bromo and chloro Suzuki coupling are two different types of reactions under C–C bond formation, Suzuki coupling. We also define a nearest neighbor baseline that adopts conditions from the most similar reactions to the query reaction within the same reaction class. Reaction similarity is defined using the Tanimoto similarity, calculated based on reaction fingerprints formed by concatenating the 2048-bit Morgan fingerprints^66^ of reactants and product(s).

The exact implementation of the two baselines varies by task. For example, in agent prediction, the popularity baseline reflects the most frequent sets of agents per reaction class, while in temperature prediction, it reflects the most common temperature per class (Fig. 2D). Overall, the two baselines aim to mimic plausible model-free approaches to propose conditions based on reaction precedents: popularity baseline captures the common chemical knowledge and nearest neighbor reflects literature searches.

### Model architecture

We implement two classes of models differing only in reaction featurization: a graph-based model using graph neural networks (GNNs), and a fingerprint-based model using feedforward neural networks (FFNs).

For the GNN models, we adapt the D-MPNN (Directed Message Passing Neural Network) architecture from Chemprop.^67^ Each reaction is represented as a molecular graph and processed through a message passing block to generate a learned reaction embedding. When agent information is required, we also embed the multi-hot agent vector and concatenate it with this reaction embedding. The combined representation is passed to a multilayer perceptron (MLP) to make task-specific predictions. For the FFN models, we encode the reaction as a binary vector by concatenating Morgan fingerprints of reactants and products. This reaction fingerprint is directly concatenated with the agent multi-hot vector.

Each stage is trained independently from scratch with its own dataset and model configuration (Fig. 2D). Here we briefly summarize the tasks. Further architectural and training details including hyperparameters are provided in Section S5.

#### Stage 1: agent prediction

We formulate agent prediction as a multi-label classification task over a fixed vocabulary. The model takes a reaction, a one-hot encoded reaction class, and a multi-hot agent vector as input. During training, we use data augmentation to encourage order invariance, by enumerating all input-target set pairs derived from the true agent set. During inference, agents are decoded autoregressively using beam search with width 10, predicting one agent at a time, conditioned on previously predicted agents. Duplicate predictions from different decoding orders are merged by summing their joint probabilities.

#### Stage 2: temperature prediction

Temperature prediction is formulated as a binned classification task with uniform binning across −100 to 200 °C. The model takes the reaction and predicted agent vector from stage 1 as input, outputting normalized probabilities over temperature bins. The model is trained using ground-truth, rather than predicted, agents (*i.e.*, using teacher forcing).

#### Stage 3: reactant amount prediction

Reactant amount prediction is formulated as a binned classification task over customized bins derived from empirical usage patterns (*e.g.*, 1.0 eq., 1.5 eq., *etc.*). This stage predicts the equivalence ratio for each reactant individually. Specifically, the model takes the reaction, the agent vector, and the fingerprint of a single reactant. The output is a single amount bin corresponding to that reactant. This process is repeated for all reactants in a reaction. Teacher forcing is also implemented here.

#### Stage 4: agent amount prediction

Following the same structure as stage 3, agent amount prediction is also formulated as a binned classification task. Agent amount bins are also chosen manually and can be adjusted across datasets. The key difference is that this model predicts over all agents in the vocabulary simultaneously, then applies the input agent vector as a mask to restrict predictions to only relevant agents. Training is performed with masked cross-entropy loss with teacher forcing to ensure the model learns to predict amounts only for agents present in the reaction.

### Evaluation

Evaluation is performed at two levels. First, we conduct individual stage analysis to assess the standalone performance of each stage without error propagation. Then we chain all the stages and perform end-to-end evaluation to simulate the model's intended deployment scenario.

For the end-to-end evaluation, we use a test set of 133 993 reactions from held-out patents for which ground-truth data were available for all four tasks. To generate a set containing multiple recommended conditions, we combine the top predictions from each stage. Specifically, we take the top 10 candidate agent sets, then for each, take the top 2 temperature, top 2 reactant amount, top 2 agent amount predictions, resulting in 80 possible combinations. These combined condition sets are ranked using a confidence score, defined as the weighted geometric average of the individual stage confidence scores. The weights are empirically selected *via* hyperparameter optimization on the validation set to maximize overall end-to-end accuracy. Details of the confidence scoring procedure can be found in Section S6.

## Results and discussion

### Predicting agent identity

**Fig. 3:**
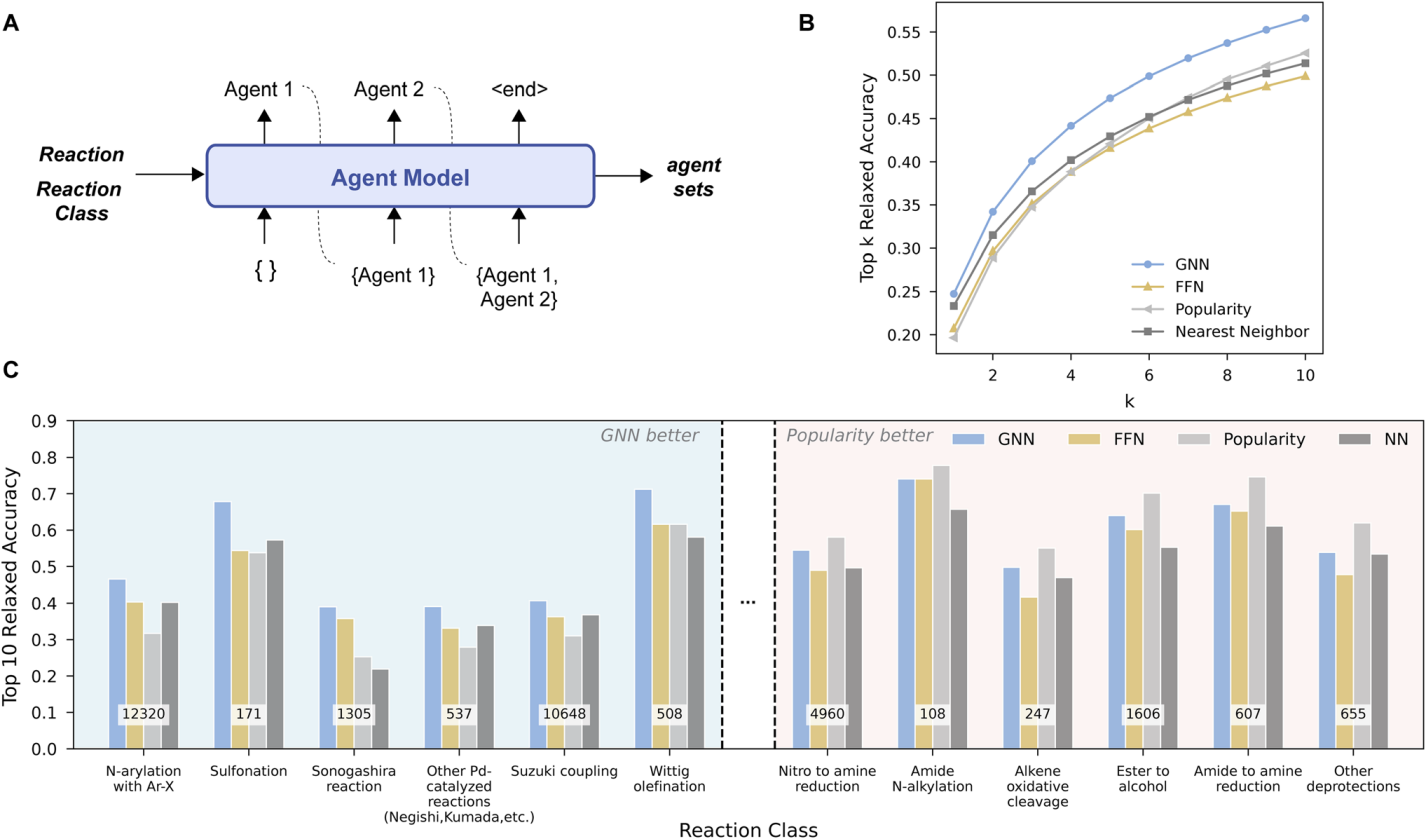
Individual stage evaluation of agent identity prediction. (A) Schematic of the agent identity model, which generates a set of agents autoregressively. (B) Top-*k* relaxed accuracy of stage 1 agent prediction. (C) Top-10 relaxed accuracy for 12 representative reaction sub-categories, selected from a total of 60 based on the performance difference between the GNN model and the popularity baseline (top 6 and bottom 6 shown). The number of test reactions per category is shown in white boxes. “Relaxed” accuracy allows predictions differing by a water molecule to count as a match due to their inconsistent presence in the dataset.

Although agent prediction is trained as a multi-label classification task, we formulate inference as an autoregressive generation process, where agents are predicted sequentially, each conditioned on the previously generated ones, until an end-of-sequence token is reached (Fig. 3A). This formulation accommodates a variable number of agents while using a fixed vocabulary of candidate agents and also allows the model to capture dependencies among agents without enforcing a fixed structure or order. Although the ground-truth agent set is unordered, the autoregressive formulation produces a sequence. To encourage order invariance, we apply data augmentation during training. Specifically, we enumerate all possible partitions of the ground-truth agent sets by splitting them into input and target sets. The model is thus trained to complete any partial agent set regardless of ordering.

We report the top-*k* accuracy, with *k* candidate agent sets generated by beam search (Fig. 3B). We used a relaxed exact match criterion, where agent sets were converted to SMILES strings and compared as unordered sets. A prediction is considered correct if the predicted and target sets match, disregarding the presence or absence of water. This exception accounts for water being inserted during agent preprocessing. A stricter index-level accuracy is also reported in Fig. S2. Under the relaxed metric, both the GNN model and the nearest neighbor baseline outperform the popularity baseline at low *k*. This suggests popularity baseline's limited coverage of reaction classes with diverse conditions (*e.g.*, cross-coupling reactions). As *k* increases, the difference between the GNN and FFN models becomes more obvious, while the popularity baseline also becomes more competitive at higher *k* values. Model performance also depends on the number of agents. As shown in Tables S14–S16, the models are biased toward shorter agent sequences, with the average predicted length falling between 2–3 agents regardless of the true number of agents. This contributes to reduced accuracy for longer sequences, alongside cumulative errors in autoregressive decoding and the scarcity of long-agent reactions.

To understand model performance across reaction types, we used the NameRxn^64^ hierarchy. Our analysis focused on the intermediate sub-category level (*e.g.*, Suzuki coupling). This provided a good balance between breadth and specificity: level 1 (*e.g.*, C–C bond formation) was too coarse (12 classes) and level 3 (*e.g.*, bromo Suzuki coupling) was too fine-grained (>1500 classes). Note that the popularity and the nearest neighbor baseline are still implemented at the most specific reaction class level. We ranked the 60 sub-categories present in the stage 1 test set by the performance gap between the GNN and popularity baseline. Fig. 3C shows the top 6 and bottom 6 sub-categories from the list, with the blue-shaded ones favoring GNN, while the red-shaded ones favoring popularity.

While the GNN model does not outperform the popularity baseline across all transformations, it demonstrates consistent advantages in sub-categories known to involve sensitive reaction conditions that typically require optimization. For example, *N*-arylation with Ar–X (including Buchwald–Hartwig amination, which is known for being sensitive to reaction conditions) showed the largest performance improvement from the GNN model. This suggests that the model can capture more nuanced substrate–condition relationships. In contrast, the popularity baseline performs well on reductions and deprotection reactions, for which a small number of robust protocols are considered broadly applicable. These trends are not simply driven by training data size. The reaction sub-categories where GNN performs well span a range of data sizes, indicating that performance improvements reflect learned chemical patterns, rather than merely being exposed to a greater number of training examples. We also examined condition diversity, estimated by (i) the number of unique agent sets and (ii) the fraction of test reactions whose agent set lies outside the top 10 most frequent sets within a class, and observed modest positive correlations with the performance gap between GNN and popularity (Section S7.3). This is consistent with the intuition that when a few protocols dominate, a popularity baseline suffices, whereas in more heterogeneous classes the learned model captures mid-frequency patterns that popularity misses.

### Predicting temperature

**Fig. 4:**
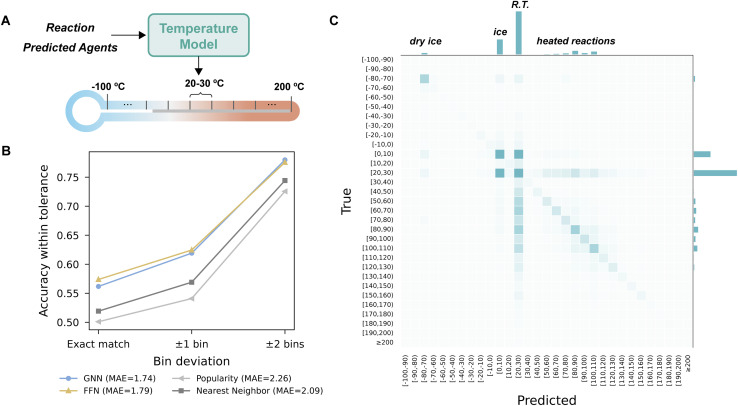
Individual stage evaluation of temperature prediction. (A) Schematic of the temperature model predicting discrete 10 °C bins. (B) Stage 2 temperature prediction accuracy under varying bin tolerances. Bin deviation reflects the distance between the predicted and true bin. The legend reports mean absolute error (MAE) in bin units. (C) Confusion matrix of the best performing FFN model, with the bin distribution for predicted and true values.

We formulate temperature prediction as a binned classification task. Regression formulations of temperature prediction fail to capture the highly irregular empirical distribution of reaction temperatures (Fig. S4). We focus on temperature ranges relevant to medicinal chemistry, ranging from −100 to 200 °C, and evenly discretize them into 10 °C increments (Fig. 4A). Given the ordinal nature of temperature labels, small deviations from the true value may still be acceptable. To account for this, we report the proportion of predictions that fall within *N* bins of the true label, along with the mean absolute error (MAE) in terms of the number of bins between the predicted and recorded bin, as measures of prediction distance.

Fig. 4B shows that GNN and FFN yield comparable performance, both outperforming the baselines with an average MAE of approximately 1.8 bins, equivalent to an error between ±20 and ±30 °C. The popularity baseline captures the common temperatures used, but is necessarily less effective for reaction classes with a wide temperature range. The nearest neighbor baseline retrieves the temperature from similar reactions from the same class but does not explicitly incorporate agent information, therefore different agents used could lead to different temperature choices. The improved performance observed in the trained models suggests that accounting for agent compatibility in addition to the substrates themselves benefits temperature prediction.

To assess whether the model captures the empirical distribution, we visualized a confusion matrix for the best-performing FFN model (Fig. 4C). The model successfully reproduced key features of the distribution. These include peaks at specific temperatures like −78 °C (dry ice), 0 °C (ice bath), room temperature, as well as a broad distribution around 80–120 °C for heated reactions. The confusion matrix showed a vertical band centered around room temperature, indicating the FFN model has a tendency to overpredict in this area. This bias is likely due to the class imbalance in the temperature dataset, where over 50% labels correspond to the room temperature bin of 20 °C to 30 °C. The FFN model also has some difficulty differentiating between ice and room temperature, likely due to their proximity, the dataset's strong imbalance, and the fact that choosing between these two temperatures sometimes reflects subtle selectivity preferences rather than clear-cut success or failure. Confusion matrices for other methods are available in Fig. S5.

### Predicting reactant amounts

**Fig. 5:**
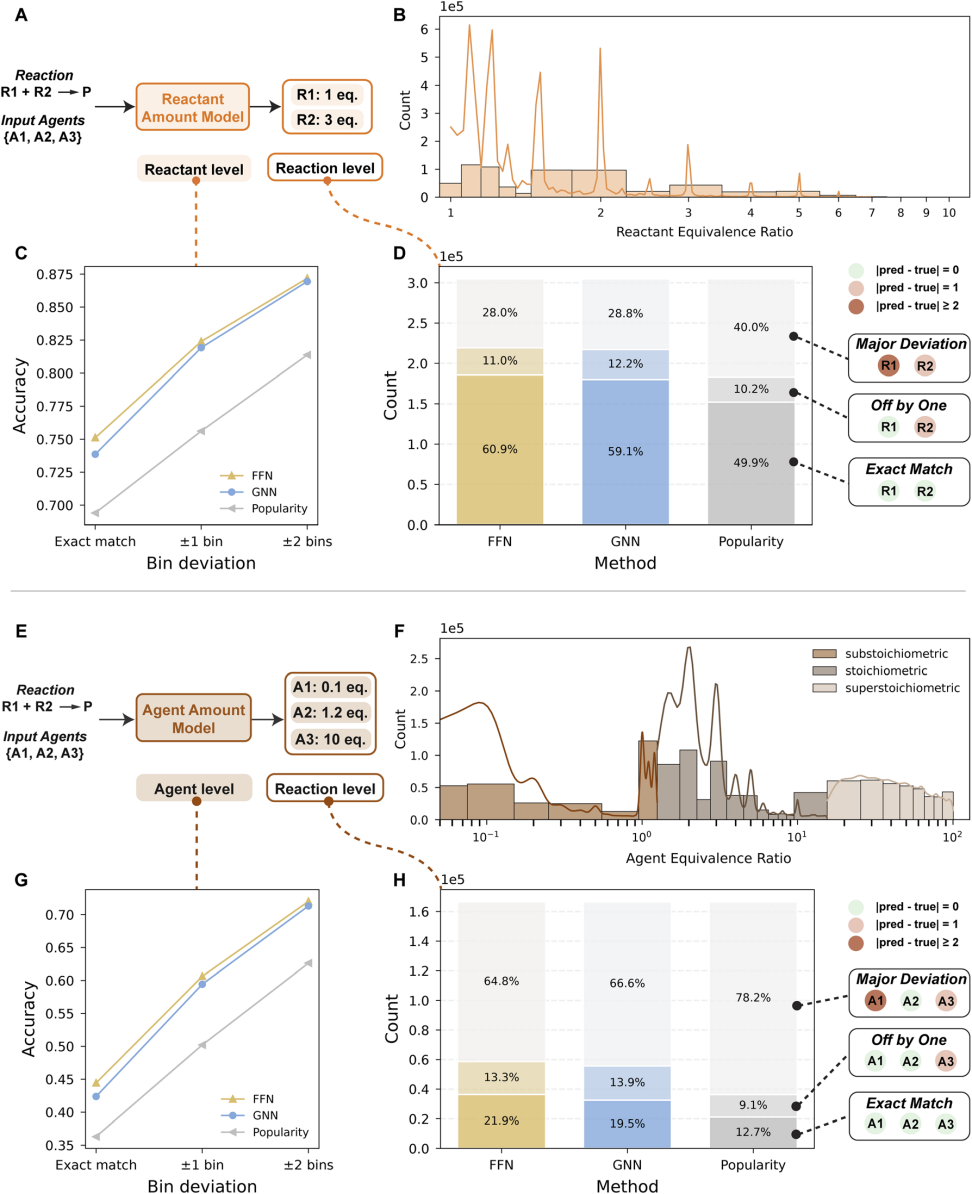
Individual stage evaluation of reactant amount and agent amount prediction. (A) Schematic of the reactant amount model predicting reactant amounts as discrete bins, evaluated at both the component and reaction levels. (B) Histogram of reactant amounts in log scale, with bin edges chosen to capture major peaks in the data. (C) Reactant-level prediction accuracy with tolerance thresholds. (D) Reaction-level accuracy categorized as follows: exact match (all reactants correctly predicted), off by one (all within ±1 bin, excluding exact), and major deviation (≥1 reactant differs by more than one bin). For each method, bars are stacked from bottom to top in this order, with darker shading indicating exact matches, medium for off-by-one and lightest for major deviation. (E–H) Repeat of panels (A–D) for stage 4 agent amount prediction using the same schemes. For (F), bin edges are chosen at substoichiometric, stoichiometric, and superstoichiometric levels to account for differences in scale between agents.

We also formulate both quantity prediction tasks as binned classification problems, analogous to the formulation used for temperature prediction. We computed the equivalence ratios, or molar ratios, of each reactant and agent relative to the limiting reactant, and observed peaks at commonly used values (*e.g.*, 1.0, 2.0, 2.5 equivalents; Fig. 5B). Therefore, instead of uniform discretization, we defined custom bins tailored to capture these frequent values in our dataset; models can be readily retrained or fine-tuned with alternative bin definitions if different levels of granularity are desired. Given the ordinal nature of the labels, we continued to evaluate performance with the off-by-*N* accuracy metric. Predictions were made per component, either a reactant or an agent, so we report both component-level accuracy and aggregate performance at the reaction level by grouping predictions across all species in a reaction.

Models predict the quantity for each reactant separately. For any given reactant, its specific fingerprint and the overall reaction context (reaction fingerprint and agents) are used to determine its amount (Fig. 5A). Fig. 5C shows the off-by-*N* accuracy, measuring the fraction of predictions within *N* bins of the true value. The popularity baseline used here is effectively a majority baseline that always predicts 1.0 equivalent (Fig. 2D). Standard baselines are less suitable for our reactant amount prediction task, as the reactant distribution is too diverse for a typical popularity baseline to be meaningful; additionally, a nearest neighbor baseline does not guarantee the same number of reactants between query and reference reactions, complicating direct comparisons. The popularity baseline predicting a constant equivalence ratio of 1.0 achieves an accuracy of nearly 70%, as by definition all reactions must have at least one limiting reactant. Further analysis excluding limiting reactants is provided in Fig. S6.

We then extended the evaluation to the reaction level by grouping predictions across all reactants (Fig. 5D). Reaction-level accuracy was categorized into three mutually exclusive buckets:

(1) Exact match: all reactants are predicted in the correct bin.

(2) Off-by-one: all predictions fall within ±1 bin of the ground truth, excluding exact matches.

(3) Major deviation: at least one reactant is off by more than one bin.

This coarse-grained breakdown simplifies evaluation by avoiding the need to enumerate all possible reactant combinations. Both the FFN and GNN models surpass the popularity baseline in exact match accuracy. The popularity baseline's notable performance of nearly 50% is partly due to the prevalence of unimolecular reactions in the test set (about 35%). The advantage of the learned models becomes more pronounced for reactions with multiple reactants. If we consider only bimolecular reactions (about 64% of the test set), the exact match accuracies are approximately 40% for the FFN model, 37% for the GNN model, but only 22% for the popularity baseline. This improvement highlights that the learned models can distinguish suitable stoichiometries in more complex scenarios. A detailed breakdown is presented in Table S18.

### Predicting agent amounts

Agent amount prediction follows a similar binned classification formulation as reactant amount. A key difference arises from the use of a fixed vocabulary for agents, allowing a model to predict amount labels for all agents simultaneously. The full vector of equivalence ratios is masked by the actual agents present in the input (Fig. 5E). Bins were selected to reflect major peaks in the data distribution. Because agents include both catalysts and solvents, which are used at substantially different scales, bins were defined to capture substoichiometric, stoichiometric, and superstoichiometric regimes (Fig. 5F). The fixed agent vocabulary enables comparison to the popularity baseline, which assigns the most frequently observed bin label for each agent. This approach reflects common usage patterns: agents typically used catalytically tend to have substoichiometric amounts, while agents typically used as solvents will have superstoichiometric amounts.

At agent level, both GNN and FFN models demonstrated comparable performance, outperforming the popularity baseline (Fig. 5G). At reaction level, the same three categories are used as shown in Fig. 5H. When comparing these results to those for reactant amount prediction, we observed a notable drop in exact matches. The decline can be attributed to two factors. First, the agent amount prediction task has more bins (27 bins) than the reactant amount task (15 bins), making it a more challenging classification task; changing the number of bins can make the quantitative accuracy arbitrarily greater or smaller. Second, reactions typically involve a higher number of distinct agents than reactants, increasing the combinatorial difficulty of predicting all quantities exactly. Despite these challenges, both models consistently outperformed the popularity baseline.

### End-to-end evaluation

To assess the practical use case of our approach, we evaluated full-pipeline performance by integrating the independently trained models into a unified inference workflow. Given a query reaction, the workflow first predicts the set of agents (stage 1). Based on the predicted agents, temperature (stage 2), reactant quantities (stage 3), and agent quantities (stage 4) are predicted in parallel. There are no hard dependencies between the last three stages. For instance, temperature predictions do not influence quantity predictions in this setup.

We evaluated QUARC's end-to-end performance on the overlapping test set under document split, where ground truth is available for all four tasks. For baselines, we considered two holistic baselines that treat the full set of conditions as a single unit rather than independently predicting each component. The popularity baseline selects the most frequently observed complete condition set (including agent identity, temperature, reactant amount and agent amount) from the training set for each reaction class. Similarly, the nearest neighbor baseline adopts the entire condition set from the most similar reactions within the same reaction class in the training data. In contrast to simply chaining most common predictions from individual stages (*e.g.*, most popular agents combined with most popular temperature), these baselines preserve the internal consistency among condition components. This matters because independently “optimal” choices may not be compatible when combined. By focusing on condition sets that have been reported in the literature as a whole, these baselines prioritize mutually compatible conditions.

We define correct conditions as exact matches across all stages: stage 1 requires exact agent matching at index level, stage 2 requires the correct temperature bin, stage 3 requires exact matches for all reactant amounts (unordered), and stage 4 requires both correct agents and quantities. For reactant amounts, since the baselines only retrieve the entire pre-existing condition sets, they cannot assign a specific quantity to each individual reactant. Our model, however, can do so based on reactant-specific fingerprints. Therefore for stage 3, we require only the frequency distribution of reactant amount bins to match, without enforcing a specific reactant-to-bin correspondence. This criterion is applied to both our models and the baselines.

**Fig. 6:**
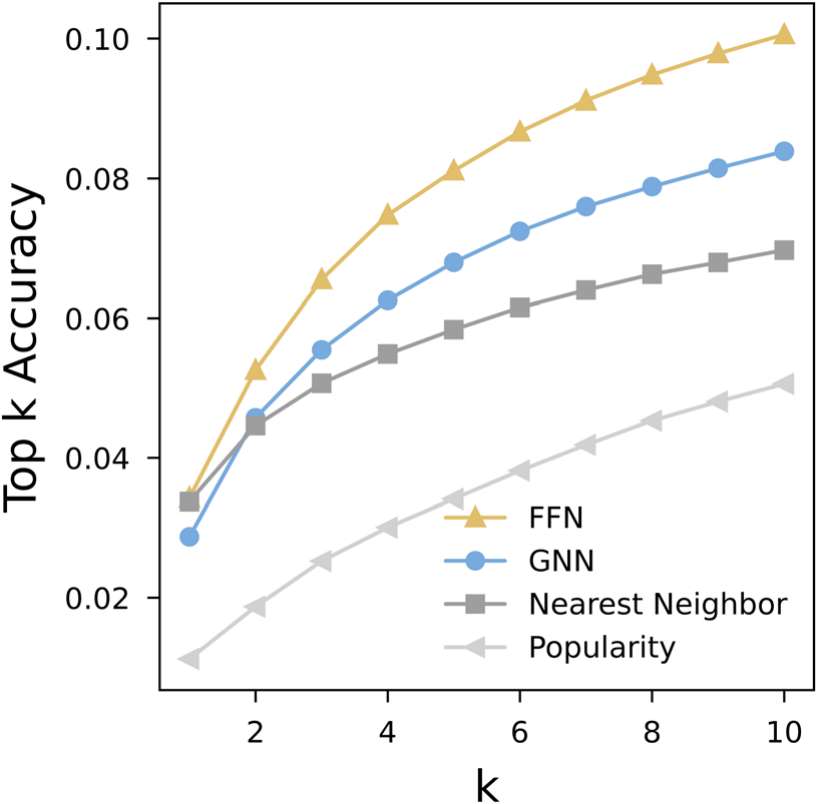
End-to-end evaluation top-*k* accuracy. To be considered correct, a complete set of conditions requires exact match at all stages. Particularly for stage 4, both agent indices and their corresponding amount labels are required to match exactly. Note that accuracy here is in terms of a strict match to literature-reported conditions, whereas in practice there are typically many possible conditions under which a reaction will proceed, particularly allowing subtle variations in equivalence ratios.

The top-*k* exact match accuracy is reported for all four methods (Fig. 6). We observed that FFN outperforms GNN, followed by the nearest neighbor and popularity baselines.

While the absolute values of top-*k* accuracy may appear modest, several factors contribute to this outcome. First, accuracy based on literature-reported conditions inherently underestimates the true accuracy of predicting chemically-viable conditions. Our evaluation metric requires an exact match to a reported condition and cannot account for plausible alternatives that could still lead to a successful reaction despite small deviations from the reported values. These “near-misses”—even something as subtle as a reactant equivalence ratio of 1.3 *versus* 1.4—may be feasible but are not captured under the current strict evaluation metric. Second, the sequential nature of the multi-stage prediction process means that errors accumulate. Inaccuracies in earlier stages, particularly in agent prediction, inevitably propagate and reduce the likelihood of recovering a fully correct condition set downstream. One possible direction to address the systematic underestimation of model performance is to evaluate predictions against the union of all reported condition sets for a reactant product pair, rather than relying on a single test record. This approach would better capture the one-to-many nature of reaction conditions. In addition, incorporating similarity-based metrics, such as those accounting for chemical similarity (*e.g.*, bases with comparable strengths) or simple set-level overlap indices, could offer a more nuanced assessment of prediction quality. Looking forward, an opportunity for future condition dataset curation lies in preserving the condition screening tables often reported by chemists, which could enable evaluation against a more comprehensive set of experimentally validated alternatives.

Although the GNN model performed better in agent prediction, the FFN model consistently showed marginal yet persistent advantages in temperature and both quantity predictions. These accumulated advantages at the later stages ultimately allowed the FFN model to achieve higher end-to-end accuracy. Importantly, both learned models substantially outperform the nearest neighbor baseline as the number of condition sets under consideration (*k*) increases, demonstrating the benefit of data-driven modeling for condition recommendation.

### Qualitative evaluation

Quantitative metrics based on the strict exact match criterion may not fully capture the practical utility of the predictions. Close approximations, particularly regarding amounts, can still be reasonable. However defining an acceptable tolerance for these close matches is challenging and must be evaluated on a case-by-base basis. In addition, occasional inaccuracies or inconsistencies in the source data can further distort evaluation results. For example, optical character recognition failures during text extraction may incorrectly parse “CuI” as “CuL” and lead to the omission of this catalyst entirely from the reaction entries, which our preprocessing steps do not rectify. “100 °C” is sometimes extracted as “100 0C”, causing our temperature parsing script to misinterpret work-up temperatures (typically, room temperature) as the ground truth reaction temperature. Here, we present several qualitative examples to allow for a direct assessment of recommended conditions.

**Fig. 7:**
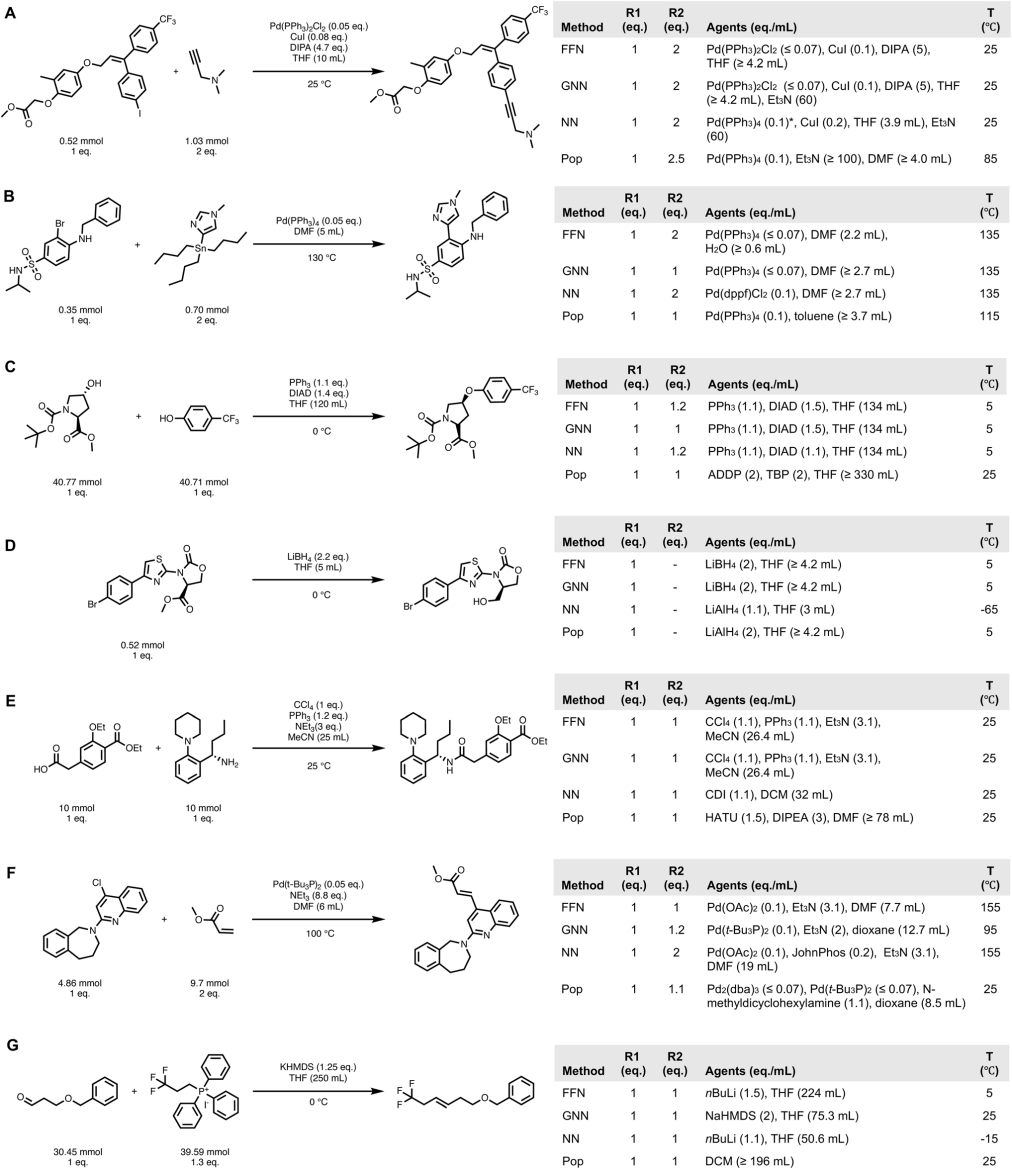
Recorded conditions and rank 1 predictions for selected reaction sub-categories (NameRxn second hierarchy) by each method. NN: nearest neighbor; Pop: popularity. For temperature, reactant amount, and agent amount predictions, values within the predicted bins are reported for clarity rather than the full intervals (*e.g.*, a temperature interval of 0 °C to 10 °C is reported as 5 °C). Agent amounts corresponding to solvents have been manually converted from equivalence ratios to volumes and scaled relative to the ground-truth limiting reactant quantity. Each sub-category includes the 95% confidence interval for FFN's win rate (%) against the NN baseline. (A) Sonogashira reaction ([79.7, 90.1]). (B) Stille reaction ([73.5, 87.6]). (C) *O*-Substitution ([64.2, 70.0]). (D) Ester to alcohol ([63.8, 76.8]). (E) *N*-Acylation to amide ([54.0, 58.6]). (F) Heck reaction ([46.9, 86.7]). (G) Wittig reaction ([42.3, 75.4]). * Manually fixed parsing errors.

We select the qualitative examples using a pair-wise win rate analysis that compares the top 10 end-to-end predictions of our models against baselines across different reaction classes grouped at the second level of the NameRxn hierarchy; we exclude instances where neither method yielded a correct prediction. Further details of win rate calculation and results are available in Section S7.9. We highlight the comparison between FFN, the top-performing model, and the nearest neighbor baseline, the strongest baseline. Fig. 7A–E show examples from reaction classes where the model demonstrates a statistically significant advantage (the lower bound of the 95% confidence interval for the win rate exceeds 50%). The final two classes (Heck and Wittig reactions) are examples where the model's advantage was less definitive, as the lower bound did not meet the 50% threshold.

Beyond individual successes, examples in Fig. 7 reveal several overarching themes about models' capacities when compared with baselines. While the nearest neighbor baseline consistently provides chemically plausible recommendations, the trained models arguably demonstrate improved substrate-awareness. By design, nearest neighbor retrieves conditions from the most similar reaction within the same reaction class, so it is rare that it would lead to an obviously poor recommendation. Indeed, many of these predictions are quite reasonable. For instance, in the Pd-catalyzed Sonogashira reaction (Fig. 7A), nearest neighbor recommended a Pd(0) complex when the reported condition used a Pd(ii) pre-catalyst system, which are functionally equivalent in this context.

However, the models can learn to recognize when the most popular conditions from a class are insufficient, particularly in terms of chemoselectivity. As Fig. 7D demonstrates, FFN and GNN appropriately select the milder reducing agent LiBH_4_ over the more aggressive LiAlH_4_ for this ester susceptible to side reactions such as oxazolidinone ring cleavage. Similarly, swapping Et_3_N for sterically hindered DIPA to suppress Glaser coupling in Sonogashira (Fig. 7A); identifying a phosphine/CCl_4_ system, a combination commonly employed in stereosensitive amide couplings, despite stereochemistry not being explicitly encoded in the input representations (Fig. 7E); choosing a non-nucleophilic base NaHMDS in a sensitive Wittig reaction to avoid side reactions with the aldehyde (Fig. 7G). These examples showcased the models' ability to capture the subtle substrate–reagent interactions that a structural distance metric (*i.e.*, Tanimoto similarity) would miss.

While seemingly minor, quantitative details beyond agent identities are also crucial to reaction outcomes, and models showed a better grasp of these fine-grained parameters than the baselines. For example, in the Mitsunobu reaction, models recommended a more robust excess of DIAD to ensure complete conversion (Fig. 7C).

These examples underscore the challenge of data quality in chemistry datasets. In the Sonogashira reaction example, we identified data fidelity issues such as missing Pd in the nearest neighbor prediction (manually added after inspection of the source patent) and missing CuI in the popularity prediction. Both mistakes are caused by optical text recognition errors and therefore difficult to rescue by a preprocessing pipeline. Baseline methods are particularly sensitive to such errors because they cannot infer missing context and average out the noise like the learned models. Addressing such data fidelity challenges may benefit from advances in reaction curation and preprocessing pipelines,^68^ as well as from emerging methods that use large language models to refine extracted reaction records.^69^

## Conclusions

We developed QUARC, a data-driven framework designed to recommend reaction conditions—including agent identity, temperature, and equivalence ratios—for diverse organic transformations. Trained on a large dataset of patent reactions, QUARC consistently outperformed two sensible chemistry-relevant baselines: a popularity baseline, which suggests the most frequently used conditions within the same reaction class, and a nearest neighbor baseline, which retrieves conditions from the closest analogous reaction within the same class. Both individual stage evaluations and end-to-end evaluation demonstrate an advantage to using the trained model. While this advantage varies across reaction types, QUARC showed advantages in predicting suitable conditions for transformations such as cross-coupling reactions where there is greater heterogeneity in reported conditions. The case studies illustrate that data-driven models are not only retrieving precedent but also leveraging reaction contexts with a nuanced understanding of selectivity, substrate constraints, and stoichiometry for condition recommendation.

QUARC can be readily integrated within CASP tools to generate conditions for hypothesized retrosynthetic pathways and thus facilitates, though does not yet directly enable, automated small molecule synthesis. Predicted conditions can serve as data-driven, literature-informed starting points for experimental optimization or expert modification.

Despite these strengths, there are several opportunities to improve QUARC in future work. First, the current encoding strategy could be more expressive. Quantities are encoded as equivalence ratios; while suitable for many reagents and catalysts, this representation is less appropriate for solvents and requires post-processing. Similarly, agents are one-hot encoded and therefore the model lacks any inductive bias as to which species are more or less functionally similar to each other. Second, ensuring reaction data are of the highest quality remains a perennial challenge. Parsing errors have led to missing or inaccurately represented critical reaction components, such as missing catalysts, ligands, or improperly grouped work-up conditions. Addressing these data inconsistencies through improved parsing methods or data curation (as more recent versions of the Pistachio dataset have implemented) will be crucial to improve the accuracy of condition models. Lastly, our current implementation involves chaining four separate models, requiring enumeration and empirically optimized ranking. Future work could explore alternative formulations capable of directly generating complete condition sets.

## Author contributions

X. S. implemented, trained, and evaluated the models and baselines. J. L. developed the preliminary data preprocessing pipeline and model architecture. X. S. and B. M. analyzed the results. All authors contributed to discussions about this project. The manuscript was drafted by X. S. with inputs from all authors. All authors reviewed and approved the final manuscript.

## Conflicts of interest

There are no conflicts to declare.

## Supplementary Material

SC-016-D5SC04957A-s001

## Data Availability

The reaction data used in this article are from the Pistachio database (2023Q2), which is a commercial resource that can be licensed from NextMove Software at https://www.nextmovesoftware.com/pistachio. The code and trained models are available at https://github.com/coleygroup/quarc. Supplementary information: Additional results, data processing details. See DOI: https://doi.org/10.1039/d5sc04957a.
