# Supplementary material for: Data-driven recommendation of agents, temperature, and equivalence ratios for organic synthesis
Source: Chem Sci. 2025 Sep 5;16(39):18176–89. doi: 10.1039/d5sc04957a (PMC12426769; doi:10.1039/d5sc04957a)
Supplement: SC-016-D5SC04957A-s001 [file SC-016-D5SC04957A-s001.pdf]

Supplementary Information (SI) for Chemical Science.
This journal is © The Royal Society of Chemistry 2025

Supplementary Information for Data-Driven Recommendation of Agents, Temperature, and Equivalence Ratios for Organic Synthesis

Xiaoqi Sun[a], Jiannan Liu[a,‡], Babak Mahjour[a], Klavs F. Jensen[a], and Connor W. Coley[a,b,*]

[a]Department of Chemical Engineering, Massachusetts Institute of Technology, 77 Massachusetts Avenue, Cambridge, MA 02139, USA.
[b]Department of Electrical Engineering and Computer Science, Massachusetts Institute of Technology, 77 Massachusetts Avenue, Cambridge, MA 02139, USA.
*E-mail: ccoley@mit.edu
‡Present address: Shanghai Institute of Organic Chemistry, Chinese Academy of Sciences, 345 Ling Ling Road, Shanghai, 200032, China.

Contents

S1 Acronyms and abbreviations 2

S2 Data preprocessing details 3

S3 Reaction class distribution 6

S4 Baselines 12
S4.1 Popularity baseline . . . 12
S4.2 Nearest Neighbor baseline . . . 12

S5 Model architectures and training details 14
S5.1 Agent identity model . . . 14
S5.2 Temperature model . . . 15
S5.3 Reactant amount model . . . 16
S5.4 Agent amount model . . . 16

S6 Details on evaluation 18

S7 Additional results 19
S7.1 Agent prediction: index-level exact match accuracy . . . 19
S7.2 Agent prediction: impact of number of agents . . . 20
S7.3 Agent prediction: diversity analysis . . . 21
S7.4 Temperature prediction: regression formulation . . . 23
S7.5 Temperature prediction: additional confusion matrices . . . 24
S7.6 Reactant amount prediction: excluding limiting reactants . . . 25
S7.7 Reaction amount prediction: breakdown of reaction-level accuracy . . . 26
S7.8 Analysis of directional deviation for binned predictions . . . 27
S7.9 Win-rate analysis . . . 28

S8 Deduplication and handling of data artifacts 33

S1 Acronyms and abbreviations

DIPA	Diisopropylamine
THF	Tetrahydrofuran
Et_3N	Triethylamine
DMF	N,N-Dimethylformamide
DIAD	Diisopropyl azodicarboxylate
ADDP	1,1'-(Azodicarbonyl)dipiperidine
TBP	Tributyl phosphate
MeCN	Acetonitrile
CDI	1,1'-Carbonyldiimidazole
DCM	Dichloromethane
HATU	O-(7-Azabenzotriazol-1-yl)-N,N,N',N'-tetramethyluronium hexafluorophosphate
DIPEA	N,N-Diisopropylethylamine
nBuLi	n-Butyllithium
NaHMDS	Sodium bis(trimethylsilyl)amide
KHMDS	Potassium bis(trimethylsilyl)amide

S2 Data preprocessing details

We extract and standardize structured reaction records from the Pistachio database, which includes metadata such as reaction SMILES and component-level quantities. Figure S1 summarizes our preprocessing pipeline:

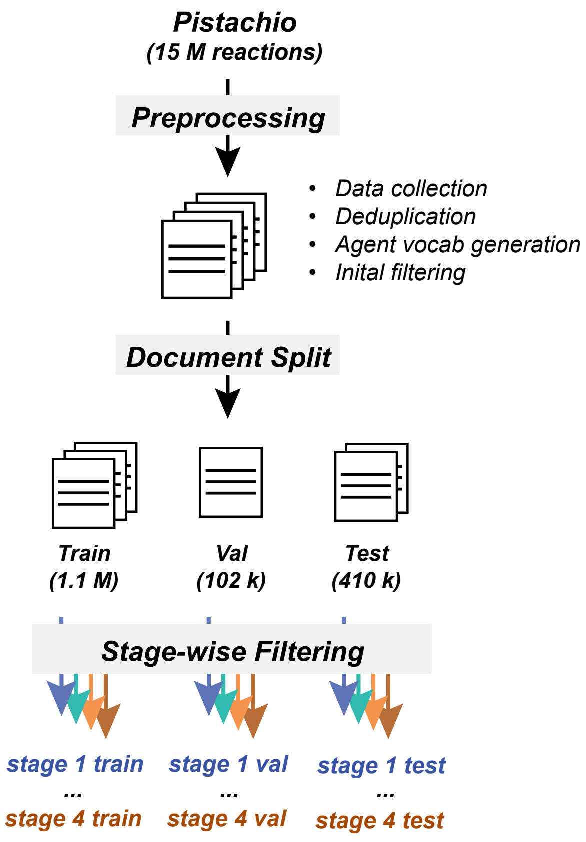

Figure S1 Data preprocessing workflow.

- **Data collection.** From approximately 15 million raw Pistachio reactions (including duplicates), we extract reaction SMILES, document ID, component roles (reactants, products, agents), and associated quantity metadata. The Pistachio records provide complete atom-mapped SMILES including all product-contributing reactants, but they are not necessarily balanced, as common leaving groups or byproducts (e.g., water from condensations) are typically omitted. In addition to reaction SMILES, each record also includes a component dictionary specifying roles, component SMILES, and quantities. These two sources are complementary: SMILES provide the reaction graph and ensure completeness, while the component dictionary provides quantities. To obtain consistent reaction entries, we reconcile the two sources through matching and validation.

  Our collection process includes the following steps:

  - *Quantity parsing.* Component-level quantities are parsed and normalized to moles. Complex mixtures and solutions (e.g., “0.5 M aqueous HCl”) are decomposed into individual constituents via a rule-based strategy to compute standardized amounts.
  - *Quality control.* Reactions are required to (i) contain at least one reactant and one product, (ii) perform a valid transformation (products not identical to reactants), and (iii) maintain disjoint reactant/agent/product sets.

– *Component reconciliation.* To align reaction SMILES with component metadata, , we perform two matching steps. Forward matching checks that parsed quantities map to molecules present in the SMILES, allowing occasional mismatches (e.g., quenching solvents). Reverse matching ensures every reactant/product in the SMILES has a corresponding entry in the component dictionary. Reaction failing these checks (e.g., due to missing or misclassified components) are discarded.

- **Deduplication.** We perform global deduplication at the *condition level*, retaining reactions with identical reactants and products but differing conditions as distinct entries.
- **Agent vocabulary generation.** To reduce sparsity, we construct a vocabulary of 1376 agents by filtering out rare species (fewer than 50 occurrences) in the deduplicated dataset. We count the frequencies of agents with valid and parsed quantity only to ensure a high-quality vocabulary. Counting frequency on all agents lead to low quality agents, including unprocessed solvent mixtures and generic entries (e.g., atmosphere, work-up conditions).
- **Initial quality filters.** We apply filters to remove low-quality or incomplete records, as described in Table S1.
- **Data splitting.** The filtered dataset is split into train, validation, and test sets with a 75:5:20 ratio at document-level. We use document split instead of random split to ensure that similar reactions – often found within the same document – are not spread across train, validation, and test sets.
- **Stage-specific filtering.** Additional filters specific to each prediction stage (Table S2) are applied to the global splits to curate stage-specific datasets. Reactions with complete information across all four stages form the overlapping subset used for end-to-end evaluation.

Full implementation details and documentation can be found in https://github.com/coleygroup/quarc. The quantity parsing framework can be extended to other structured or semi-structured sources (e.g., text-mined extraction).

Table S1 Initial filtering criteria applied during data preprocessing.

**Filter Category**	**Criterion**	**Rationale**
Component count	Product count = 1<br>$1 \leq$ Reactant count ≤ 5<br>$0 \leq$ Agent count ≤ 5	Focus on single-product reactions and excludes overly complex reactions
Molecule size	≤ 50 atoms for reactants<br>≤ 50 atoms for products	Focus on reasonably-sized molecules
Reaction Quality	Remove rare agents<br>Reaction must have temperature or agents<br>RDKit parsability for full and individual SMILES	Validate reactions have valid and sufficient information
Solvent Validation	Common solvent in reactants or agents	Ensure Substrates should be dissolved. (optional, $\sim$20% removal)
Reaction Classification	Remove uncategorized reactions (NameRxn = 0.0)	Ensure only training with high-quality reactions (optional, $\sim$20% removal)

Table S2 Stage-specific filtering criteria for individual prediction tasks.

Stage	Filter Name	Description
Stage 1	Agent existence	At least one agent must be present
	Unique agents	All agents must be unique
	Agent amount existence	All agents must have quantity information (Keep only high-quality agents)
Stage 2	Temperature existence	Temperature must be specified
	Temperature range	Temperature must lie between -100 °C and 200 °C
Stage 3	Unique reactants	All reactants must be unique
	Reactant amount existence	All reactants must have quantity information
	Reactant ratio range	Molar ratios of reactants must be within 0 to 7 (90th percentile)
Stage 4	Agent existence	At least one agent must be present
	Agent amount existence	All agents must have quantity information
	Unique agents	All agents must be unique
	Agent ratio range	Agent molar ratios (relative to limiting reactant) must be within 0.001 to 1000
	Non-solvent high ratio	Non-solvent agents must have a molar ratio upper bound of 10

The resulting spitted data for each stage is reported in Table S3.

Table S3 Final dataset sizes following a document split after all preprocessing steps. * indicates additional deduplication was applied.

	Train	Val	Test
Stage 1	793838	52088	186064*
Stage 2	1162172	77228	311225
Stage 3	1139485	76208	304838
Stage 4	626884	41395	166516
End-to-end	506223	33419	133993

An additional deduplication is necessary for the isolated evaluation of stage 1. The global deduplication operates at full condition level, meaning reactions with identical agents but different temperatures are treated as unique entries and could exist in both train and test sets. While valid for end-to-end evaluation, this could allow the agent identity model to be tested on an agent set it has already seen in the training set. Therefore, we removed any test entry whose ground truth agents is recovered with a closest neighbor with a Tanimoto distance of 1.

S3 Reaction class distribution

Here we report the distribution of reaction sub-categories (NameRxn 2nd hierarchy) in each data split for all five stages (Stages 1–4 and the end-to-end evaluation). The stage-specific filters applied can alter the reaction class distribution, but the overall pattern is roughly consistent across all five stages. Within each table, the top 40 sub-categories ranked by test split percentage are shown individually, and all remaining are aggregated as the "Other" category.

Table S4 Distribution of reaction sub-categories for Stage 1.

**Reaction Sub-category**	**Count**			**Percentage (%)**		
	Train	Val	Test	Train	Val	Test
N-acylation to amide	93860	6952	23335	11.82	13.35	12.54
Other functional group interconversion	58250	3924	13393	7.34	7.53	7.2
O-substitution	57848	3898	12419	7.29	7.48	6.67
N-arylation with Ar-X	50971	3535	12320	6.42	6.79	6.62
NH deprotections	43713	2916	10799	5.51	5.6	5.8
Suzuki coupling	45228	2999	10648	5.7	5.76	5.72
Heteroaryl N-alkylation	38925	2471	9388	4.9	4.74	5.05
RCO2H deprotections	35020	2384	8749	4.41	4.58	4.7
Reductive amination	33164	1971	7303	4.18	3.78	3.92
Nitro to amine reduction	19979	1276	4960	2.52	2.45	2.67
N-containing heterocycle formation	20767	1293	4909	2.62	2.48	2.64
Other C-C bond formation	19433	1271	4529	2.45	2.44	2.43
ROH deprotections	17764	1256	3863	2.24	2.41	2.08
Halogenation	18450	1070	3797	2.32	2.05	2.04
N-acylation to urea	15383	806	3757	1.94	1.55	2.02
O-acylation to ester	14240	881	3399	1.79	1.69	1.83
NH protections	15416	965	3389	1.94	1.85	1.82
N-sulfonylation	14933	1031	3264	1.88	1.98	1.75
N-substitution with alkyl-X	10929	696	2390	1.38	1.34	1.28
Alcohol to halide	10246	596	2362	1.29	1.14	1.27
Carbamate/carbonate formation	9623	596	2343	1.21	1.14	1.26
Alcohols to aldehydes	9860	623	2251	1.24	1.2	1.21
S-substitution	8668	544	2147	1.09	1.04	1.15
Alkene to alkane	8904	589	2071	1.12	1.13	1.11
O-containing heterocycle formation	8036	613	1938	1.01	1.18	1.04
Other reductions	7787	472	1845	0.98	0.91	0.99
Oxidations at sulfur	7403	489	1778	0.93	0.94	0.96
Other organometallic C-C bond formation	7424	398	1693	0.94	0.76	0.91
O-sulfonylation	7844	451	1609	0.99	0.87	0.86
Ester to alcohol	6893	395	1606	0.87	0.76	0.86
Sonogashira reaction	5446	273	1305	0.69	0.52	0.7
Ketone to alcohol	5432	311	1161	0.68	0.6	0.62
ROH protections	5097	335	1106	0.64	0.64	0.59
Stille reaction	4236	258	1060	0.53	0.5	0.57
S-containing heterocycle formation	3786	196	946	0.48	0.38	0.51
Grignard reaction	4502	258	940	0.57	0.5	0.51
Other functional group addition	3414	261	770	0.43	0.5	0.41
Other heteroatom alkylation/arylation	2702	232	765	0.34	0.45	0.41

Table S4 continued from previous page

Reaction Sub-category	Count			Percentage (%)		
	Train	Val	Test	Train	Val	Test
Cyano or imine to amine	3036	150	738	0.38	0.29	0.4
Other oxidations	3281	195	713	0.41	0.37	0.38
Other	35945	2258	8306	4.53	4.33	4.46

Table S5 Distribution of reaction sub-categories for Stage 2.

Reaction Sub-category	Count			Percentage (%)		
	Train	Val	Test	Train	Val	Test
N-acylation to amide	130442	9451	35680	11.22	12.24	11.46
Other functional group interconversion	90788	6102	24247	7.81	7.9	7.79
O-substitution	88037	5886	22756	7.58	7.62	7.31
N-arylation with Ar-X	77083	5287	20758	6.63	6.85	6.67
NH deprotections	76938	5057	19769	6.62	6.55	6.35
Suzuki coupling	58667	4064	15388	5.05	5.26	4.94
Heteroaryl N-alkylation	53843	3484	15106	4.63	4.51	4.85
RCO2H deprotections	53537	3652	14939	4.61	4.73	4.8
Reductive amination	45605	2772	11551	3.92	3.59	3.71
Other C-C bond formation	33730	2261	9293	2.9	2.93	2.99
Halogenation	29310	1768	7634	2.52	2.29	2.45
N-containing heterocycle formation	27710	1827	7476	2.38	2.37	2.4
ROH deprotections	29431	2131	7327	2.53	2.76	2.35
Nitro to amine reduction	22596	1445	6336	1.94	1.87	2.04
O-acylation to ester	20748	1296	5583	1.79	1.68	1.79
NH protections	19860	1251	5409	1.71	1.62	1.74
N-acylation to urea	19371	1008	5320	1.67	1.31	1.71
N-sulfonylation	19340	1272	4885	1.66	1.65	1.57
N-substitution with alkyl-X	15281	997	3991	1.31	1.29	1.28
Alcohol to halide	14304	881	3800	1.23	1.14	1.22
Carbamate/carbonate formation	13011	783	3592	1.12	1.01	1.15
O-containing heterocycle formation	12682	1217	3582	1.09	1.58	1.15
Alcohols to aldehydes	13324	833	3576	1.15	1.08	1.15
Other organometallic C-C bond formation	13306	748	3546	1.14	0.97	1.14
S-substitution	12605	853	3490	1.08	1.1	1.12
Other reductions	12697	817	3465	1.09	1.06	1.11
Ester to alcohol	12164	714	3260	1.05	0.92	1.05
Oxidations at sulfur	10700	731	2890	0.92	0.95	0.93
O-sulfonylation	10325	614	2801	0.89	0.8	0.9
Alkene to alkane	9548	632	2490	0.82	0.82	0.8
Ketone to alcohol	7921	450	2025	0.68	0.58	0.65
ROH protections	7418	484	1980	0.64	0.63	0.64
Sonogashira reaction	7360	405	1955	0.63	0.52	0.63
Grignard reaction	7112	475	1829	0.61	0.62	0.59
Other functional group addition	6256	420	1587	0.54	0.54	0.51
Stille reaction	5273	340	1553	0.45	0.44	0.5
S-containing heterocycle formation	4436	269	1396	0.38	0.35	0.45
Other heteroatom alkylation/arylation	3767	302	1326	0.32	0.39	0.43
Other oxidations	4803	307	1266	0.41	0.4	0.41
Friedel-Crafts reaction	4459	305	1262	0.38	0.39	0.41
Other	56384	3637	15106	4.85	4.71	4.85

Table S6 Distribution of reaction sub-categories for Stage 3.

Reaction Sub-category	Count			Percentage (%)		
	Train	Val	Test	Train	Val	Test
N-acylation to amide	127228	9412	34456	11.17	12.35	11.3
Other functional group interconversion	84296	5679	22691	7.4	7.45	7.44
N-arylation with Ar-X	75995	5391	20375	6.67	7.07	6.68
O-substitution	76854	5044	19496	6.74	6.62	6.4
NH deprotections	73768	4973	19322	6.47	6.53	6.34
RCO2H deprotections	59312	4082	16766	5.21	5.36	5.5
Suzuki coupling	61758	4186	15984	5.42	5.49	5.24
Heteroaryl N-alkylation	52152	3288	14309	4.58	4.31	4.69
Reductive amination	41996	2536	10463	3.69	3.33	3.43
Nitro to amine reduction	30861	1990	8596	2.71	2.61	2.82
Other C-C bond formation	30478	2090	8541	2.67	2.74	2.8
ROH deprotections	33463	2381	8330	2.94	3.12	2.73
Halogenation	30407	1840	7967	2.67	2.41	2.61
N-containing heterocycle formation	27446	1866	7632	2.41	2.45	2.5
N-acylation to urea	18776	1036	5418	1.65	1.36	1.78
NH protections	19458	1193	5257	1.71	1.57	1.72
N-sulfonylation	19183	1236	4802	1.68	1.62	1.58
O-acylation to ester	15736	1016	4372	1.38	1.33	1.43
Alcohols to aldehydes	14373	961	3846	1.26	1.26	1.26
Other reductions	13908	881	3805	1.22	1.16	1.25
N-substitution with alkyl-X	13892	900	3562	1.22	1.18	1.17
O-containing heterocycle formation	12514	1188	3500	1.1	1.56	1.15
Alkene to alkane	13405	861	3491	1.18	1.13	1.15
Ester to alcohol	12745	754	3459	1.12	0.99	1.13
S-substitution	12526	833	3420	1.1	1.09	1.12
Alcohol to halide	11997	708	3156	1.05	0.93	1.04
Carbamate/carbonate formation	11498	722	3147	1.01	0.95	1.03
Other organometallic C-C bond formation	11411	668	3078	1.0	0.88	1.01
Oxidations at sulfur	9848	684	2637	0.86	0.9	0.87
O-sulfonylation	9630	580	2568	0.85	0.76	0.84
Ketone to alcohol	8388	501	2183	0.74	0.66	0.72
Sonogashira reaction	7391	414	1978	0.65	0.54	0.65
ROH protections	7138	477	1869	0.63	0.63	0.61
Stille reaction	5528	374	1562	0.49	0.49	0.51
S-containing heterocycle formation	5212	296	1557	0.46	0.39	0.51
Other functional group addition	5741	386	1452	0.5	0.51	0.48
Cyano or imine to amine	5618	324	1445	0.49	0.43	0.47
Other deprotections	4989	271	1382	0.44	0.36	0.45
Amide to amine reduction	4828	373	1374	0.42	0.49	0.45
Other heteroatom alkylation/arylation	3535	275	1251	0.31	0.36	0.41
Other	54203	3538	14339	4.76	4.64	4.7

Table S7 Distribution of reaction sub-categories for Stage 4.

Reaction Sub-category	Count			Percentage (%)		
	Train	Val	Test	Train	Val	Test
N-acylation to amide	72168	5406	19753	11.51	13.06	11.86
O-substitution	47236	3157	11971	7.54	7.63	7.19
Other functional group interconversion	44925	2970	11767	7.17	7.17	7.07
N-arylation with Ar-X	41753	2947	11306	6.66	7.12	6.79
Suzuki coupling	38128	2562	9895	6.08	6.19	5.94
Heteroaryl N-alkylation	30579	1995	8231	4.88	4.82	4.94
RCO2H deprotections	26581	1838	7491	4.24	4.44	4.5
NH deprotections	28318	2009	7489	4.52	4.85	4.5
Reductive amination	25993	1485	6212	4.15	3.59	3.73
N-containing heterocycle formation	16864	1077	4612	2.69	2.6	2.77
Nitro to amine reduction	16401	1028	4591	2.62	2.48	2.76
Other C-C bond formation	16049	1084	4508	2.56	2.62	2.71
Halogenation	15590	906	3939	2.49	2.19	2.37
NH protections	13427	832	3472	2.14	2.01	2.09
N-acylation to urea	11735	639	3362	1.87	1.54	2.02
ROH deprotections	13349	899	3327	2.13	2.17	2.0
N-sulfonylation	12002	832	2931	1.91	2.01	1.76
O-acylation to ester	10873	693	2917	1.73	1.67	1.75
Carbamate/carbonate formation	7582	478	2193	1.21	1.15	1.32
Alcohol to halide	7808	447	2124	1.25	1.08	1.28
N-substitution with alkyl-X	8420	552	2117	1.34	1.33	1.27
Alcohols to aldehydes	7664	484	1968	1.22	1.17	1.18
S-substitution	6971	444	1929	1.11	1.07	1.16
O-containing heterocycle formation	6559	502	1832	1.05	1.21	1.1
Alkene to alkane	7182	488	1810	1.15	1.18	1.09
Other reductions	6783	405	1805	1.08	0.98	1.08
O-sulfonylation	6574	379	1714	1.05	0.92	1.03
Ester to alcohol	6169	352	1669	0.98	0.85	1.0
Other organometallic C-C bond formation	6070	321	1644	0.97	0.78	0.99
Oxidations at sulfur	5476	369	1490	0.87	0.89	0.89
Ketone to alcohol	4618	261	1183	0.74	0.63	0.71
Sonogashira reaction	4712	228	1183	0.75	0.55	0.71
ROH protections	4485	302	1149	0.72	0.73	0.69
Stille reaction	3571	225	1019	0.57	0.54	0.61
S-containing heterocycle formation	3276	172	987	0.52	0.42	0.59
Grignard reaction	3262	196	802	0.52	0.47	0.48
Other heteroatom alkylation/arylation	2259	197	731	0.36	0.48	0.44
Other functional group addition	2670	213	687	0.43	0.51	0.41
Cyano or imine to amine	2468	118	671	0.39	0.29	0.4
Amide to amine reduction	2037	180	590	0.32	0.43	0.35
Other	28297	1723	7445	4.51	4.16	4.47

Table S8 Distribution of reaction sub-categories for end-to-end evaluation.

Reaction Sub-category	Count			Percentage (%)		
	Train	Val	Test	Train	Val	Test
N-acylation to amide	58938	4490	15894	11.64	13.44	11.86
N-arylation with Ar-X	34834	2413	9419	6.88	7.22	7.03
Other functional group interconversion	35881	2359	9347	7.09	7.06	6.98
O-substitution	36253	2350	9082	7.16	7.03	6.78
Suzuki coupling	33721	2285	8781	6.66	6.84	6.55
Heteroaryl N-alkylation	24363	1661	6773	4.81	4.97	5.05
RCO2H deprotections	22209	1527	6321	4.39	4.57	4.72
NH deprotections	20369	1510	5233	4.02	4.52	3.91
Reductive amination	19597	1096	4755	3.87	3.28	3.55
Other C-C bond formation	13164	880	3631	2.6	2.63	2.71
N-containing heterocycle formation	12385	799	3396	2.45	2.39	2.53
Halogenation	13543	789	3362	2.68	2.36	2.51
Nitro to amine reduction	11527	713	3169	2.28	2.13	2.37
NH protections	11877	743	3054	2.35	2.22	2.28
ROH deprotections	10886	702	2694	2.15	2.1	2.01
N-acylation to urea	9518	482	2625	1.88	1.44	1.96
N-sulfonylation	9836	715	2414	1.94	2.14	1.8
O-acylation to ester	7866	498	2153	1.55	1.49	1.61
Alcohol to halide	6570	358	1796	1.3	1.07	1.34
Alcohols to aldehydes	6641	393	1734	1.31	1.18	1.29
N-substitution with alkyl-X	6920	466	1715	1.37	1.39	1.28
Carbamate/carbonate formation	5942	350	1712	1.17	1.05	1.28
Other reductions	5835	339	1548	1.15	1.01	1.16
Ester to alcohol	5627	326	1540	1.11	0.98	1.15
O-sulfonylation	5767	315	1528	1.14	0.94	1.14
S-substitution	5702	373	1511	1.13	1.12	1.13
O-containing heterocycle formation	5347	411	1473	1.06	1.23	1.1
Other organometallic C-C bond formation	5282	279	1394	1.04	0.83	1.04
Oxidations at sulfur	4596	296	1276	0.91	0.89	0.95
Alkene to alkane	4904	339	1190	0.97	1.01	0.89
Sonogashira reaction	4046	200	1044	0.8	0.6	0.78
ROH protections	4033	261	1041	0.8	0.78	0.78
Ketone to alcohol	4126	227	1037	0.82	0.68	0.77
Stille reaction	3045	200	907	0.6	0.6	0.68
S-containing heterocycle formation	2185	133	729	0.43	0.4	0.54
Grignard reaction	2703	155	656	0.53	0.46	0.49
Other heteroatom alkylation/arylation	1901	175	616	0.38	0.52	0.46
Other functional group addition	2290	185	575	0.45	0.55	0.43
Amide to amine reduction	1691	150	507	0.33	0.45	0.38
Cyano or imine to amine	2051	90	498	0.41	0.27	0.37
Other	22252	1386	5863	4.4	4.15	4.38

S4 Baselines

S4.1 Popularity baseline

The popularity baseline predicts conditions based on their frequency of occurrence within the training data for a given reaction context. The specific implementation varies by prediction task. Standard library functions (e.g., Python's `collections.Counter.most_common())` are used for frequency counting and to resolve any ties in frequency.

- **Agent Prediction:** For this task, reaction classes are defined by their complete NameRxn[?] classification (e.g., 3.1.1 representing bromo Suzuki coupling, which falls under sub-category 3.1 Suzuki coupling, and category 3 C-C bond formation). Within each such reaction class, we count the frequency of entire agent sets (encompassing agents) observed in the training data. At inference, for a query reaction belonging to a specific class, the baseline returns the top-k most frequently occurring agent sets from that class's counted list. If a reaction class has insufficient training examples to generate a meaningful top-k list (e.g., for k=10), fewer predictions or none may be returned for that specific query.

- **Temperature Prediction:** For each reaction class, again defined by its complete NameRxn classification, we count the frequency of temperature, categorized into discrete bins as discussed in the main text. For top-k accuracy, the baseline returns the bins with the k-th highest frequencies for that reaction class.

- **Reactant Amount Prediction:** Direct frequency counting of reactant amounts is impractical due to the high diversity of reactants. Instead, this baseline leverages majority baseline of limiting reactants. Since we express amounts as molar ratios, where at least one reactant is typically assigned as 1.0 equivalent. This baseline therefore uniformly predicts 1.0 equivalent for all reactants.

- **Agent Amount Prediction:** For each distinct agent, we count the frequency of the amount bins at which it was used. For top-k accuracy, given an agent, the baseline predicts the k amount bins most frequently associated with that specific agent in the training data.

- **End-to-end Prediction:** When chaining everything together, we count the frequency of the entire set condition, including the agents, temperature, reactant amount and agent amount, with all numerical values binned. This baseline then returns the k-th full set as its predictions.

S4.2 Nearest Neighbor baseline

The nearest neighbor (NN) baseline identifies reactions in the training set most similar to a given query reaction and uses their recorded conditions as predictions. Reaction similarity is computed as the Tanimoto similarity between reaction fingerprints, defined as the concatenated 2048-bit Morgan fingerprints (radius 3) of reactants and products, generated using RDKit's `MorganGenerator` (`rdFingerprintGenerator.GetMorganGenerator(radius=3, fpSize=2048)`).

The nearest neighbor search is conducted using the `faiss` package[1] for efficiency. The the Jaccard metric (equivalent to the Tanimoto definition) is used. We adopt a "local search" strategy: for each query reaction, neighbors are sought only from training reactions within the same specific reaction class. Compared to the global approach of searching within the entire training set, this is approach is much faster computationally and improves performance by removing less relevant reactions using different transformations. The local search is the primary nearest neighbor baseline referenced in the main text.

The implementation of the nearest neighbor baseline is to adopt the predictions from k closest reactions:

- **Agent Prediction:** Take the complete agent sets from the top-k nearest reactions as the top-k predictions.

- **Temperature Prediction:** From the top-k neighbors, determine the most likely temperature bin by majority vote. (This approach is used because for temperature evaluation we compare the top-1 prediction to the target within a tolerance, so voting over similar bins is preferred over just relying on a single neighbor).
- **End-to-end Prediction:** Adopt the entire set of conditions (with proper binning) from the k-th closest neighbor to use as the k-th prediction.

Note that for the individual stage evaluation of reactant and agent amount, we exclude the nearest baseline, because even if chemically similar, neighbors are not guaranteed to share the same number of reactants and the same type of agents. A one-to-one mapping between the reactants in the query reaction and the neighbor reaction requires proper clustering. Therefore for individual stage analysis of reactant amount and agent amount where we focus on component-level and reaction-level accuracies, we exclude the nearest neighbor baseline. For the end-to-end evaluation, we relax the correctness criterion by disregarding the direct correspondence between a specific reactant in the query and neighbor reactions. For instance, if a Suzuki coupling neighbor used 1.0 equivalent of an aryl halide and 1.2 equivalents of a boronic acid, the nearest neighbor baseline would predict the set of amounts 1.0 eq., 1.2 eq.. A prediction is considered correct if the distribution of predicted reactant amount bins matches the distribution of true reactant amount bins for the query reaction, irrespective of which reactant is assigned which amount. This allows the nearest neighbor baseline to be included in the end-to-end evaluation, albeit with slightly overoptimistic results. We justify this overoptimistic reporting as the nearest neighbor model is serving as a baseline.

S5 Model architectures and training details

This section provides the detailed description of model our Graph Neural Network (GNN) and Feedforward Neural Network (FFN) models. The main difference between these two model architectures lies in their respective reaction representations. The GNN models use molecular graphs with message-passing to generate learned embeddings. In contrast, the FFN models use reaction fingerprints (concatenating Morgan fingerprints of reactants and products) as binary vector inputs.

The GNN architecture used for all tasks comprises three main components: reaction encoder, auxiliary input encoders, and prediction heads.

- **Reaction encoder.** The reaction encoder is chemprop's `CondensedGraphOfReactionFeaturizer` with the `REAC_DIFF` mode.[?] Atom-mapped reaction SMILES strings are converted to condensed graphs of reaction and the graphs are processed through directed message-passing neural networks (D-MPNNs) and mean-pooled into a learned reaction embedding. We use the atom mapping provided in Pistachio without modification.
- **Auxiliary input encoders.** Each task may supply additional categorical signals that are first embeded by simple MLPs (`Linear`, `LayerNorm`, `ReLU`). For instance, the input agents are represented as a 1376-dim multi-hot vector, which is projected to a 512-dim dense embedding. These encoders are to avoid combining the learned graph embedding with binary vectors.
- **Prediction heads.** The reaction graph embedding and any auxiliary embeddings are concatenated and fed into the stage-specific prediction heads, which are multi-layer FFNs with residual connections. All heads share the same backbone of input projection, n residual blocks, and a task-specific read-out.

The FFN architecture is similar to that of GNN on a higher-level. Reaction details and auxiliary inputs (e.g., input agent multi-hot vector, reaction class one-hot vectors) are combined and then fed into task-specific prediction heads.

- **Reaction encoder.** The reaction encoder has no learning involved. For each reaction, it generates a 2048-bit Morgan Fingerprint with the `rdkit.Chem.rdMolDescriptors.GetMorganFingerprintAsBitVect()` method with a radius of 3 for reactants and products respectively. Then a 4096-bit reaction fingerprint is constructed by concatenating reactant and products fingerprints.
- **Prediction heads.** Both the reaction fingerprints and auxiliary inputs are binary, so no embedding is need. Instead, all binary vectors are first concatenated and fed into the individual prediction heads, which are also multi-layer FFNs with residual connections.

S5.1 Agent identity model

The agent prediction task is formulated as an autoregressive generation problem, where the model predicts agents sequentially until the stop token is emitted. During training, the task is posed as a multi-label classification problem, with the objective being "given the input agents (multi-hot), predicting the remaining agents (multi-hot)". However, during inference, beam search decoding with a beam width of 10 is used to sequentially generate the agent set.

Data augmentation. Agent identity prediction is essentially a set prediction, but in our task formulation, we treat it as a sequence modeling task (using searching algorithm to decode). To encourage agent order invariance, we use data augmentation to enumerate all possible binary partitions, a input set and a target set. For each reaction having N agents, 2^N training examples are created. To avoid bias towards reactions with more agents, we apply sample weights derived from Pascal-triangle coefficients, assigning lower weights to larger subsets. An example is shown in Table S9.

Training. The model was trained using a sampled-weighted cross-entropy loss. This was chosen over standard BCE loss for multi-label classification for its better empirical performance for our multi-label classification tasks, potentially because it better aligns with the sequential decoding used at inference. Early stopping monitors greedy search exact match accuracy on the validation set (patience = 5).

Table S9 Example data augmentation for a reaction with ground truth agent {A,B,C}. The inverse of Pascal-triangle coefficients is used as weights for each group of $\binom{3}{k}$

Input agent set	Target agent set	Weight
$\varnothing$	$\{A,B,C\}$	1
$\{A\}$<br>$\{B\}$<br>$\{C\}$	$\{B,C\}$<br>$\{A,C\}$<br>$\{A,B\}$	$\frac{1}{3}$
$\{A,B\}$<br>$\{A,C\}$<br>$\{B,C\}$	$\{C\}$<br>$\{B\}$<br>$\{A\}$	$\frac{1}{3}$
$\{A,B,C\}$	`<EOS>`	1

Inference. Agent set predictions are generated using beam search decoding with a beam size of 10. At each step, the algorithm maintains the top-10 partial sequences based on their joint sequence probabilities. Since the task is to predict unordered agent sets, different generation sequences resulting in the same set of agents are merged by summing their beam scores. Decoding for a given beam terminates when all active sequences either predict an end-of-sequence token (`<EOS>`) or reach a maximum step of 6. Predictions are only considered complete upon generation of an `<EOS>` token.

Hyperparameter. Hyperparameter optimization was performed with Ray Tune[2] using 20 trials. Hyperparameters are reported in Table S10.

Table S10 Agent identity model hyperparameters. Values marked * were fixed ones.

Model	Parameter	Search space	Best
GNN	Message-passing depth	{2, 3, 4, 5, 6}	2
	Graph hidden dim	{256, 512, 1024}	1024
	FFN hidden dim	{1024, 2048}	2048
	Learning rate	$10^{[-8,-4]}$	2.3×10^{-4}
	FFN layers	3*	–
FFN	FFN hidden dim	{512, 1024, 2048}	2048
	FFN layers	{2,3,4}	3
	Learning rate	$10^{[-8,-4]}$	2.3×10^{-4}
	FP radius	3*	–

S5.2 Temperature model

Model architecture. Temperature prediction is formulated as multiclass classification task over discrete bins. Input features include the reaction representation (either a reaction fingerprint or a learned GNN embedding) and agents info (a multi-hot agent vector, either predicted or ground truth agent sets). The output is a softmax distribution over temperature bins (32 bins).

Hyperparameter. Hyperparameters are reported in Table S11.

Table S11 Temperature model hyperparameters. Values marked * were fixed.

Model	Parameter	Search space	Best
GNN	Message-passing depth	{2, 3, 4, 5, 6}	6
	Graph hidden dim	{256, 512, 768, 1024}	768
	Learning rate	$10^{[-9,-4]}$	9.2×10^{-5}
	FFN hidden dim	2048*	–
	FFN layers	3*	–
FFN	FFN hidden dim	{256, 512, 1024, 2048}	2048
	FFN layers	{2, 3, 4, 5, 6}	6
	Learning rate	$10^{[-8,-4]}$	1.1×10^{-5}
	FP radius	3*	–

S5.3 Reactant amount model

Recap of problem setup: individually generate amount for each reactant (using a shared encoder with operations to augment/multiplex) and update weights in a batch, just draw it out. The output reaction

Model architecture. Reactant amount prediction is implemented using a shared-weight FFN that predicts amounts for each reactant individually. For each reactant, the model takes three inputs: (1) the global reaction representation (fingerprint or learned graph embedding), (2) the input agent vector (multi-hot vector), and (3) the fingerprint of the specific reactant. These are combined and passed through the FFN to produce a prediction over reactant amount bins (15 bins). The model is applied independently to each reactant using shared weights, enabling fine-grained, per-reactant predictions.

Hyperparameter. Hyperparameters are reported in Table S12.

Table S12 Reactant amount model hyperparameters. Values marked * were fixed.

Model	Parameter	Search space	Best
GNN	Message-passing depth	{2, 3, 4, 5}	3
	Graph hidden dim	{256, 512, 1024}	512
	Learning rate	$10^{[-8,-3]}$	7.6×10^{-5}
	FFN hidden dim	2048*	–
	FFN layers	3*	–
FFN	FFN hidden dim	{256, 512, 1024, 2048}	2048
	FFN layers	{2, 3, 4}	2
	Learning rate	$10^{[-9,-4]}$	8.6×10^{-5}
	Activation	{ReLU, LeakyReLU}	ReLU
	FP radius	3*	–

S5.4 Agent amount model

Model architecture. The agent amount prediction model employs a one-shot prediction architecture that simultaneously predicts binned amounts for all possible agents in a single forward pass. The agent amount prediction head receives concatenated reaction fingerprints and agent input vectors. The output layer is reshaped to produce a tensor of shape (batch size, 1376 agents, 27 agent amount bins). This one-shot design enables parallel prediction across the full vocabulary, with each agent receiving its own predicted distribution over amount bins. During training, a mask is applied to ignore predictions for agents not present in the reaction.

Hyperparameter. Hyperparameters are reported in Table S13.

Table S13 Agent amount model hyperparameters. Values marked * were fixed.

Model	Parameter	Search space	Best
GNN	Message-passing depth	{2, 3, 4, 5}	4
	Graph hidden dim	{256, 512, 1024}	512
	Learning rate	$10^{[-8,-3]}$	$6.4 \times \times 10^{-5}$
	FFN hidden dim	2048*	–
	FFN layers	3*	–
FFN	FFN hidden dim	{256, 512, 1024, 2048}	2048
	FFN layers	{2, 3, 4}	3
	Learning rate	$10^{[-8,-4]}$	1.1×10^{-5}
	Activation	{ReLU, LeakyReLU}	ReLU
	FP radius	3*	–

S6 Details on evaluation

To generate end-to-end predictions for a full set of reaction conditions, we developed a framework that integrates the four individual models. The process involves a hierarchical enumeration of candidate conditions, followed by a final re-ranking step to identify the most probable complete set.

The prediction process is sequential. First, the model for Stage 1 generates the 10 most likely sets of reaction agents. For each of these 10 agent sets, the Stage 2 model then predicts the two most probable reaction temperatures, Stage 3 model predicts two sets of reactant amount values, and Stage 4 model predicts two sets of agent amount values. Since both amount prediction models predict probabilities for each chemical component individually. To form a coherent set of amounts (e.g., for all reactants), we first generate combinations from the top individual predictions for each component. These combinations are then ranked to select the top-2 overall sets. This ranking is based on a stage-specific confidence score, defined as the geometric mean of the individual component probabilities. This approach normalizes for the number of components, ensuring that predictions for reactions with more reagents are not unfairly penalized. The full enumeration results in a total of 80 unique, complete sets of reaction conditions (10 × 2 × 2 × 2) that are then evaluated and ranked.

Each step in the generation process is guided by a confidence score. The final ranking of the 80 complete condition sets is determined by an Overall Confidence Score, which combines the scores from each of the four stages. These scores are defined as:

- **Stage 1** confidence is defined by the beam search score, which is the joint probability of each sequence of agents. Sequences with the same set of agents but in different order are merged by score addition.
- **Stage 2** uses the softmax probability of the predicted temperature bin.
- **Stages 3 and 4** assign per-component probabilities, and the score for the full set is computed as the geometric mean of the top predictions across all reactants or agents.

$$\text{Reactant Amount Score: } S_3 = \left(\prod_{i=1}^{n_R} p_i\right)^{\frac{1}{n_R}}$$

$$\text{Agent Amount Score: } S_4 = \left(\prod_{i=1}^{n_A} p_i\right)^{\frac{1}{n_A}}$$

where n_R and n_A refer to number of reactants and number of agents respectively.

The overall confidence score for a complete set of conditions is calculated as the weighted geometric mean of the four stage-specific scores:

$$\text{Overall Confidence Score} = \left(\prod_{i=1}^{4} S_i^{w_i}\right)^{1/\sum_{i=1}^{n} w_i}$$

Here S_i refers to score for stage i, and w_i refers to the corresponding weight. These weights were tuned via hyperparameter optimization on a validation set to balance the contribution of each model to the final prediction.

S7 Additional results

S7.1 Agent prediction: index-level exact match accuracy

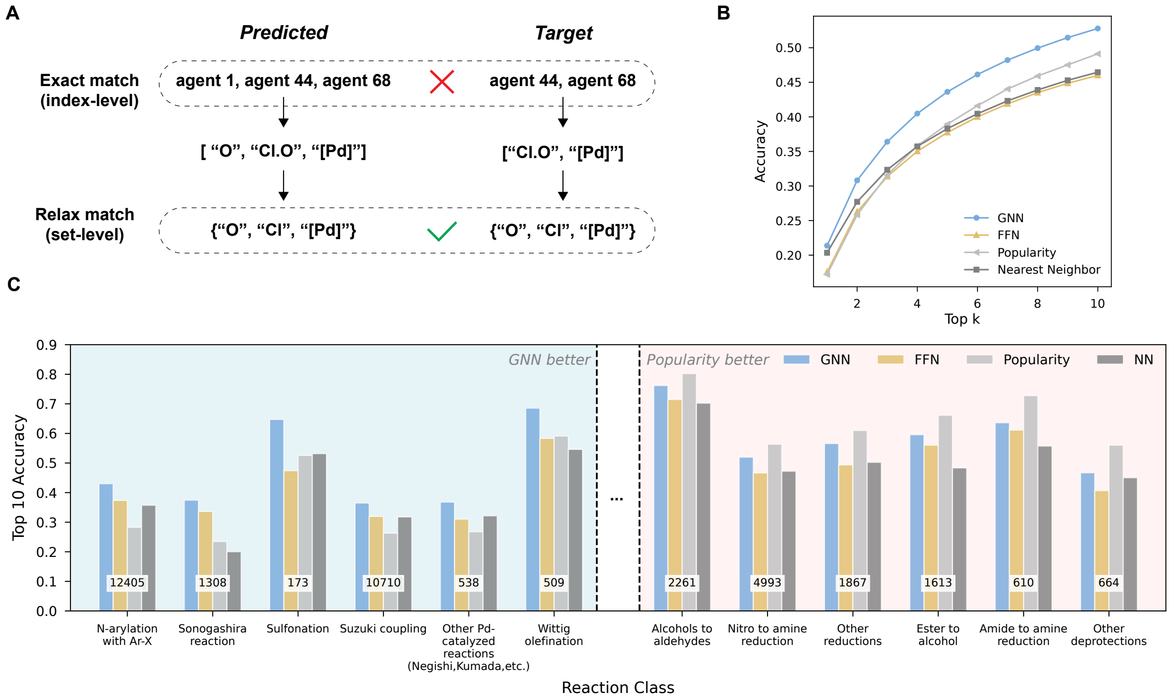

Figure S2 Stage 1 top-k accuracy using the exact match index accuracies. (A) Schematic illustrating the difference between the exact and the relaxed correctness criterion. (B) Top-k exact accuracy of stage 1 agent prediction. (C) Top-10 exact accuracy for 12 representative reaction sub-categories, selected from 60 based on GNN vs. popularity baseline performance gap (top 6 and bottom 6 shown). Blue shading: GNN favored; red: popularity favored. Test reactions per category in white boxes.

The manuscript evaluates agent prediction using a relaxed, set-level accuracy, which focuses on comparing the set of SMILES fragments rather than agent indices, while also allowing differences in the presence/absence of water to account for its frequent mistaken inclusion from workup conditions. This section presents results using a stricter index-level exact match criterion, where the predicted sequence of agent indices must perfectly match the target sequence, as illustrated in Figure S2A. This provides a more stringent test of model performance.

Figure S2B shows the overall top-k index-level exact match accuracy. Consistent with the relaxed metric results reported in the main text, the GNN model generally outperforms other models (FFN, Popularity, Nearest Neighbor) across k=1 to 10. Figure S2C shows top-10 index-level exact match accuracy for 12 representative reaction sub-categories, selected to highlight where GNN performs best and worst relative to the Popularity baseline. The GNN's varying advantages across reaction types (e.g., strong in N-arylation, less so in Alcohols to aldehydes compared to Popularity) mirror trends observed with the relaxed metric.

Overall, the relative performance of models and their strengths/weaknesses within specific reaction classes remain consistent between this stricter index-level evaluation and the relaxed set-level evaluation in the main text. Minor variations in test reaction counts per category can occur due to a final deduplication filter whose precise output is sensitive to the matching criterion.

S7.2 Agent prediction: impact of number of agents

Table S14 Top-k accuracy for agent prediction broken down by methods and number of agents.

# Agents	FFN (%)			GNN (%)			Nearest Neighbor (%)			Popularity (%)			# Reactions
	Top 1	Top 5	Top 10	Top 1	Top 5	Top 10	Top 1	Top 5	Top 10	Top 1	Top 5	Top 10	
1	33.9	60.5	70.3	34.7	63.3	74.9	33.2	55.6	65.2	26.5	62.4	75.2	34557
2	23.1	46.1	55.3	27.6	52.5	62.6	25.2	47.3	56.9	24.4	49.9	60.8	73439
3	14.2	31.9	39.5	18.7	38.2	46.5	18.6	35.8	43.4	14.4	30.9	40.3	48956
4	11.1	26.0	31.8	16.8	32.9	39.1	15.8	30.6	36.4	9.8	20.0	29.4	23755
5	6.1	15.2	19.4	11.0	22.3	26.8	12.1	21.5	25.1	1.6	4.2	8.1	5357

To further investigate the effect of sequence length on autoregressive agent prediction, we evaluated predicted sequence lengths against the gold number of agents (Table S15 - S16). For each length, we report the following metrics:

- Average predicted length among top 10 predictions
- Fraction of reactions where the top 1 prediction has the correct length,
- Fraction of reactions where the top 10 predictions contain a correct-length candidate
- Fraction of Top-10 predictions (10 * number of reactions) with correct length.

Both GNN and FFN tend to generate around two agents on average, regardless of the true number of agents. While beam search seems to improve the coverage by including correct-length predictions in top 10 predictions, those candidates are rarely ranked first. This suggest the current confidence-based ranking doesn't fully compensate for the models' bias towards shorter sequences. Future work could address this limitation through alternative scoring strategies that better balance sequence length.

Table S15 GNN predicted sequence length analysis.

# Agents	Avg Pred Length	% Top-1 Correct Length	% Reactions with Correct Length in Top-10	% Predictions with Correct Length in Top-10
1	1.78 ± 0.45	61.3	95.0	37.0
2	2.24 ± 0.48	62.2	98.8	48.3
3	2.69 ± 0.62	42.5	94.9	38.1
4	3.21 ± 0.66	44.5	83.6	33.1
5	3.45 ± 0.71	19.7	53.8	15.1

Table S16 FFN predicted sequence length analysis.

# Agents	Avg Pred Length	% Top-1 Correct Length	% Reactions with Correct Length in Top-10	% Predictions with Correct Length in Top-10
1	1.77 ± 0.43	62.0	94.3	34.3
2	2.19 ± 0.49	58.4	98.7	51.1
3	2.59 ± 0.63	36.6	91.1	37.1
4	3.11 ± 0.71	35.6	75.4	30.5
5	3.33 ± 0.77	13.5	43.8	12.4

S7.3 Agent prediction: diversity analysis

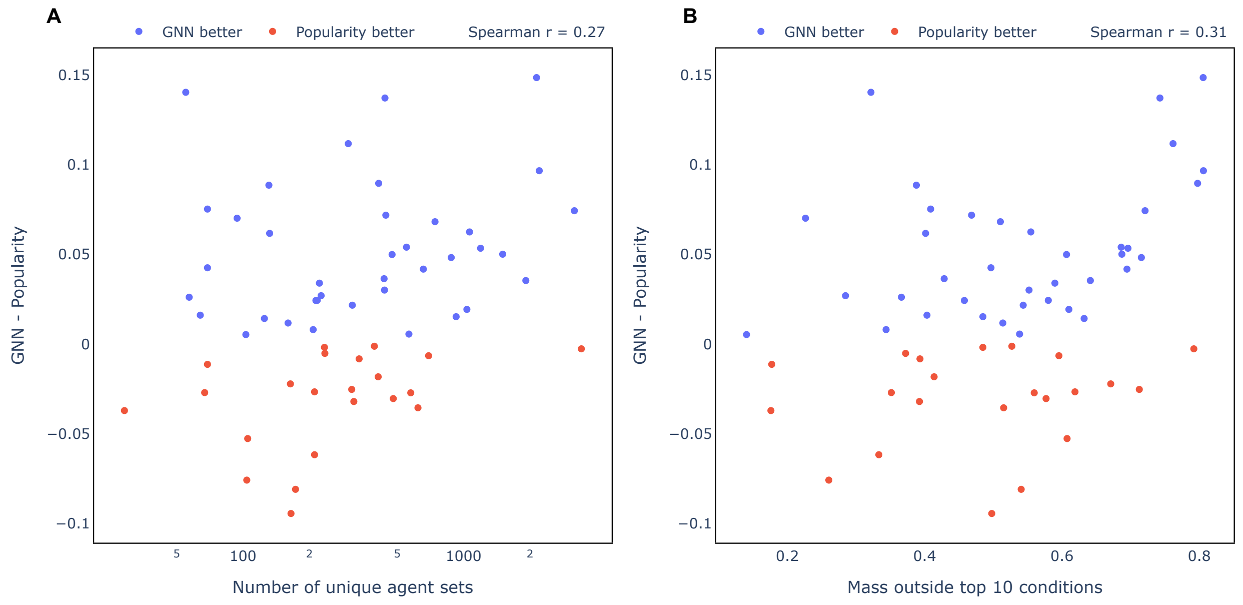

Figure S3 GNN - popularity performance difference v.s. diversity metrics for each reaction sub-category (NameRxn 2nd hierarchy). (A) Number of unique agent sets. A weak positive Spearman correlation (ρ = 0.27) suggests that GNN tends to perform better than the popularity baseline in classes with broader agent usage. (B) Tail mass (fraction of test reactions that fall outside the 10 most frequent agent sets in each sub-category). A similar weak positive correlation (ρ = 0.31) indicates that GNN performs better than the popularity baseline in classes where its agent distribution is less concentrated among the top 10 most frequent sets.

Table S17 Number of unique agent sets and tail mass per reaction sub-category (NameRxn 2nd hierarchy).

Reaction Sub-category	Δ (GNN - Pop.)	# Reactions	# Unique Agent Sets	Tail Mass
N-arylation with Ar-X	0.15	12320	2142	0.81
Sulfonation	0.14	171	55	0.32
Sonogashira reaction	0.14	1305	440	0.74
Other Pd-catalyzed reactions (Negishi,Kumada,etc.)	0.11	537	300	0.76
Suzuki coupling	0.10	10648	2203	0.81
Wittig olefination	0.09	508	131	0.39
Other functional group addition	0.09	770	412	0.80
Nitration	0.08	186	69	0.41
Halogenation	0.07	3797	742	0.51
N-acylation to urea	0.07	3757	444	0.47
N-acylation to amide	0.07	23335	3179	0.72
Oxidations at nitrogen	0.07	570	94	0.23
Heteroaryl N-alkylation	0.06	9388	1064	0.55
Amidine formation	0.06	486	132	0.40
Other C-C bond formation	0.05	4529	1194	0.70
Reductive amination	0.05	7303	1504	0.69

Table S17 continued from previous page

Reaction Sub-category	Δ (GNN - Pop.)	# Reactions	# Unique Agent Sets	Tail Mass
S-substitution	0.05	2147	551	0.69
O-acylation to ester	0.05	3399	879	0.72
Carbamate carbonate formation	0.05	2343	474	0.61
RCO2H protections	0.04	141	69	0.50
O-substitution	0.04	12419	1915	0.64
O-containing heterocycle formation	0.04	1938	657	0.69
N-sulfonylation	0.04	3264	436	0.43
Other protections	0.03	153	57	0.37
Other heteroatom alkylation/arylation	0.03	765	222	0.59
Oxidations at sulfur	0.03	1778	226	0.28
N-substitution with alkyl-X	0.03	2390	438	0.55
Stille reaction	0.02	1060	313	0.54
S-containing heterocycle formation	0.02	946	214	0.46
NH deprotections	0.02	10799	925	0.48
N-containing heterocycle formation	0.02	4909	1035	0.61
Cyano or imine to amine	0.02	738	217	0.58
Nitrile to acid	0.02	248	64	0.40
RCO2H deprotections	0.01	8749	565	0.54
O-sulfonylation	0.01	1609	208	0.34
Friedel-Crafts reaction	0.01	510	160	0.51
Other acylation	0.01	280	125	0.63
Grignard reaction	0.01	940	103	0.14
Other organometallic C-C bond formation	-0.0	1693	394	0.53
Other functional group interconversion	-0.0	13393	3419	0.79
ROH protections	-0.0	1106	234	0.48
ROH deprotections	-0.01	3863	694	0.60
Alkene to alkane	-0.01	2071	336	0.39
Ketone to alcohol	-0.01	1161	235	0.37
Acid to acid chloride	-0.01	621	69	0.18
Heck reaction	-0.02	316	164	0.67
Alcohol to halide	-0.02	2362	410	0.41
Other reductions	-0.03	1845	480	0.58
Other oxidations	-0.03	713	311	0.71
Dehydration	-0.03	564	211	0.62
Alkyne to alkane	-0.03	222	67	0.35
NH protections	-0.03	3389	576	0.56
Alcohols to aldehydes	-0.03	2251	318	0.39
Amide N-alkylation	-0.04	108	29	0.18
Nitro to amine reduction	-0.04	4960	621	0.51
Alkene oxidative cleavage	-0.05	247	105	0.61
Ester to alcohol	-0.06	1606	211	0.33
Amide to amine reduction	-0.08	607	104	0.26
Other deprotections	-0.08	655	173	0.54
Salt formation	-0.09	625	165	0.50

S7.4 Temperature prediction: regression formulation

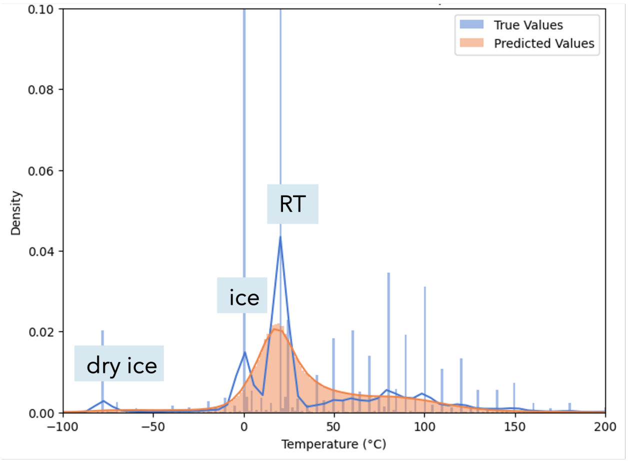

Figure S4 Distribution of true and predicted temperature using the regression formulation.

Before using the binned classification formulation for temperature prediction, we initially considered direct regression. Figure S4 shows the distribution of true (blue) and predicted (orange) reaction temperatures in the test set. The true data clearly shows a multi-modal distribution, with prominent peaks at common temperatures like dry ice, ice, and room temperature. However, the regression model's predictions form a largely unimodal distribution, failing to capture these distinct, chemically relevant temperature modes and instead smoothing over them. This inability of direct regression to adequately represent the multi-modal nature of the temperature data motivated our adoption of the binned classification approach, which more effectively learns these common temperature categories.

S7.5 Temperature prediction: additional confusion matrices

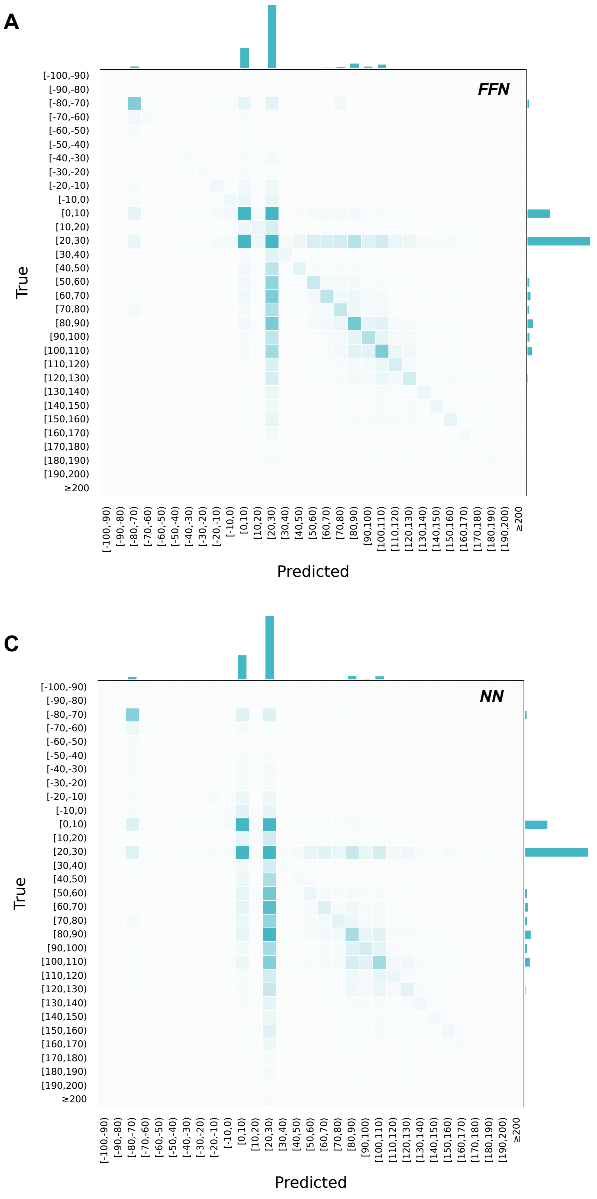

Figure S5 Confusion matrices for all methods. (A) FFN. (B) GNN. (C) Nearest neighbor. (D) Popularity. To better highlight the differences between FFN and GNN, the colormap of the heatmap is capped at a maximum value of 8000. Panel (A) is identical to Fig. 2C in the main text, but the reduced colormap threshold results in enhanced contrast.

S7.6 Reactant amount prediction: excluding limiting reactants

The main manuscript notes that 1.0 equivalents is the most frequent reactant amount, forming a strong majority class. To assess model performance beyond this dominant case, Figure S6 evaluates reactant amount prediction accuracy on a test subset that excludes all ground truth instances where the reactant amount is 1.0 equivalent. This effectively removes the "limiting reactant" cases from the evaluation.

As expected, when 1.0 equivalent instances are excluded, a baseline that always predicts "1.0 Eq." achieves zero exact match accuracy (Figure S6). For further comparison, a baseline predicting the next most frequent class (1.1 Eq) is also shown. The figure demonstrates that while overall accuracies for all models decrease on this more challenging subset, both the FFN and GNN models maintain a clear performance advantage over these baselines, even when allowing for deviations of ±1 or ±2 bins. This result supports the conclusion that the FFN and GNN models have learned meaningful patterns for predicting reactant amounts beyond simply memorizing the most frequent 1.0 equivalent value.

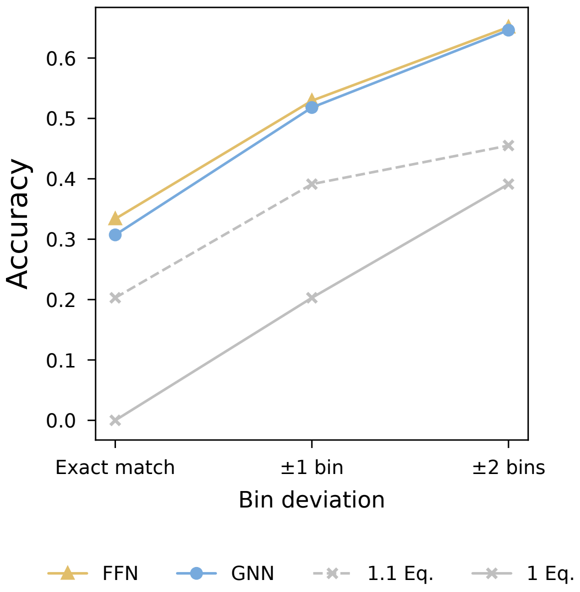

Figure S6 Reactant-level accuracy with tolerance for stage 3 reactant amount prediction, excluding all reactants with 1.0 equiv (i.e., limiting reactants) as ground truth. The 1.0 eq-only baseline drops to zero under this setting, confirming its reliance on class imbalance. For reference, the performance of a 1.1 eq-only baseline is also shown as the next most frequent class. While overall accuracy decreases, both the FFN and GNN models continue to outperform the baselines.

S7.7 Reaction amount prediction: breakdown of reaction-level accuracy

Table S18 Reaction-level accuracy for reactant amount prediction broken down by methods and number of reactants.

# Reactants	**FFN (%)**			**GNN (%)**			**Popularity (%)**			# Reactions
	Exact Match	Off by One	Major Deviation	Exact Match	Off by One	Major Deviation	Exact Match	Off by One	Major Deviation	
1	100.0	0.0	0.0	100.0	0.0	0.0	100.0	0.0	0.0	107257
2	39.9	17.1	43.1	37.0	18.8	44.2	22.8	15.8	61.5	195036
3	27.9	14.7	57.4	26.3	15.6	58.1	15.6	9.9	74.4	2456
4	11.2	5.6	83.1	8.9	15.7	75.3	3.4	0.0	96.6	89

S7.8 Analysis of directional deviation for binned predictions

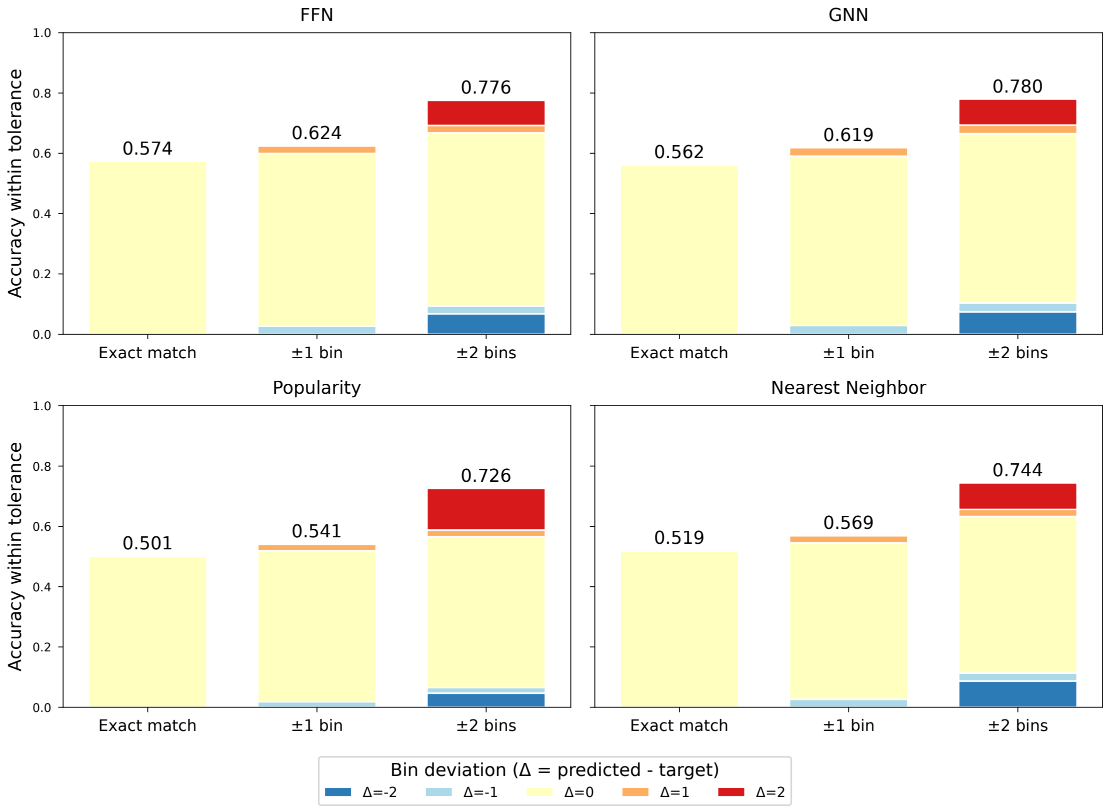

Figure S7 Directional contributions for binned temperature predictions. FFN, GNN, and nearest neighbor models show balanced ±1/±2 deviations, while the popularity baseline exhibits a strong overpreidction bias.

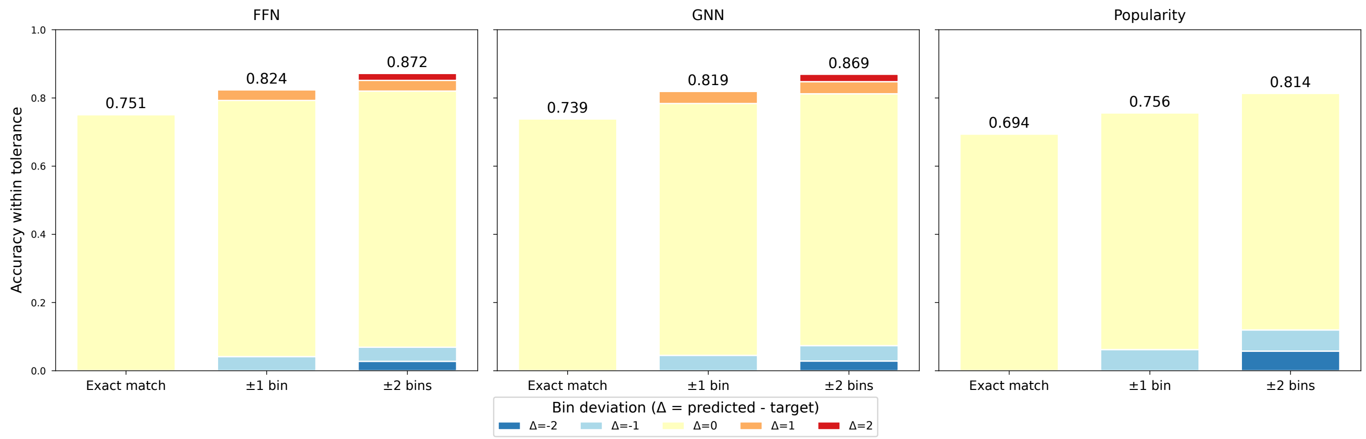

Figure S8 Directional contributions for binned reactant amount predictions. All models show a slight skew toward underprediction, with the popularity baseline consistently predicting lower than the true bin.

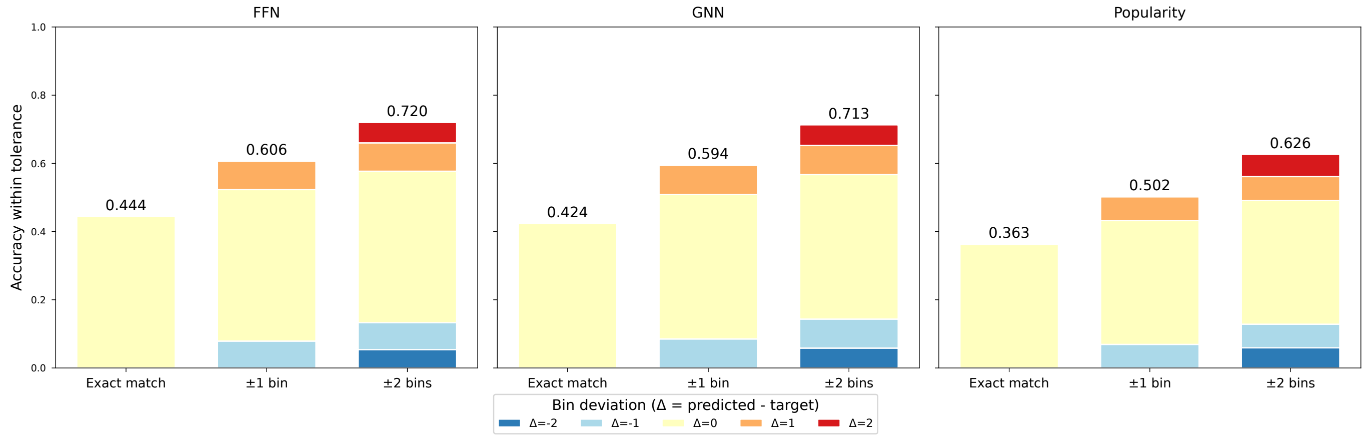

Figure S9 Directional contributions for binned agent amount predictions. All models exhibit similar patterns with mild upward skew, and no substantial differences in deviation trends.

S7.9 Win-rate analysis

For the overlapping test set (130k reaction) we generated top-10 ranked predictions by each method and grouped by second-level NameRxn classes. For each class, we compared between pairs of models and baselines.

- **Wins** Correct predictions appeared at higher rank (i.e., lower index) in model's prediction compared to the baseline.
- **Losses** are cases when baseline ranked the correct prediction higher.
- **Ties** include both true ties (identical ranks) and null ties, where neither method produced a correct prediction in the top-10; the latter accounted for over 80% of reactions in a class.
- **Win-rate** is calculated as $N_{wins}/(N_{wins}+N_{losses})$, with all ties excluded.

We computed 95% confidence intervals on the win rate using the Wilson score interval for binomial proportions. While we performed this analysis across all pairwise combinations of models (FFN, GNN) and baselines (nearest neighbor, popularity), we focus our discussion primarily on the strongest-performing model (FFN) against the most competitive baseline (nearest neighbor), as this comparison best highlights model improvements. Figure S10, Figure S11, Figure S12, and Figure S13 report the win rates and their confidence intervals for each model-baseline pair.

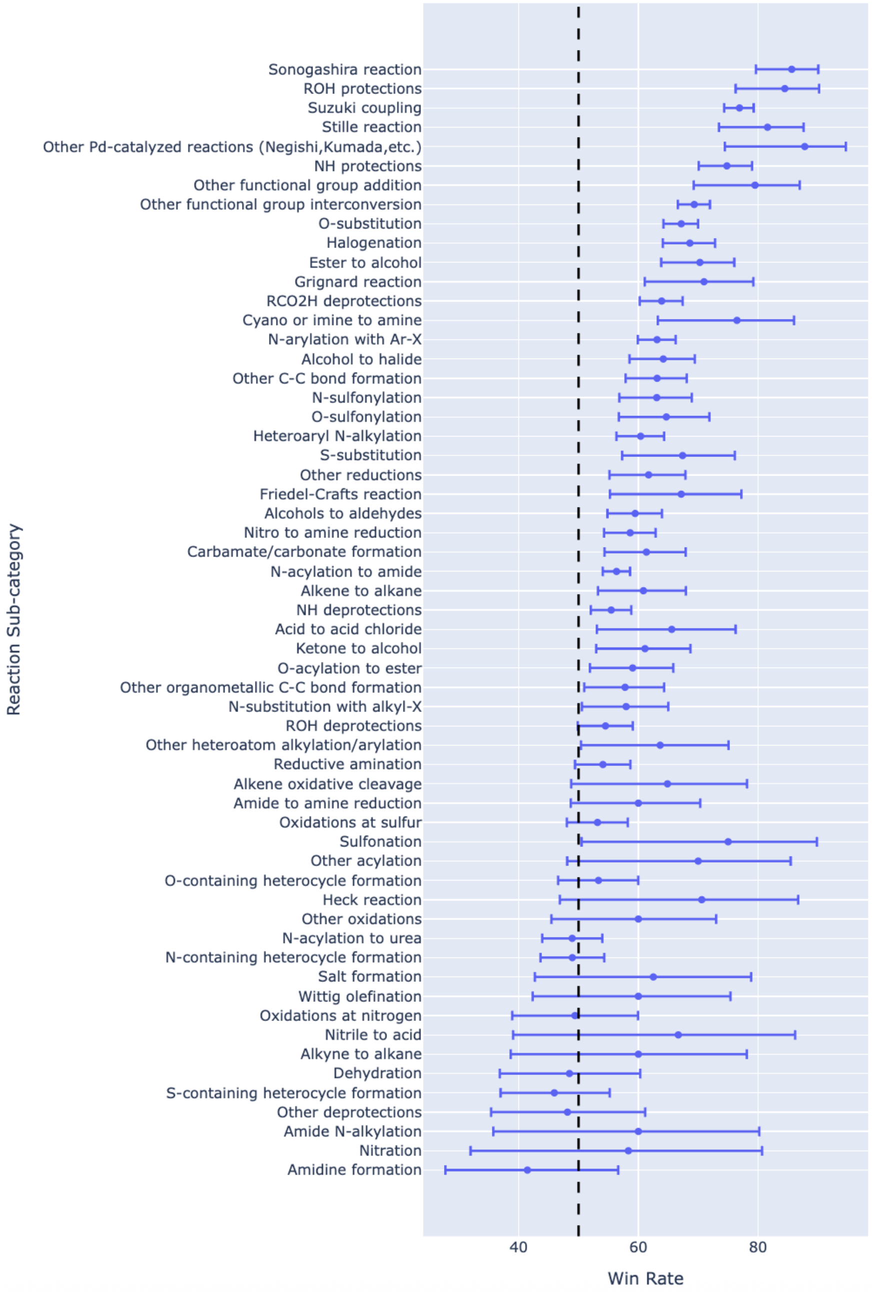

Figure S10 Win-rate analysis comparing FFN and nearest neighbor baseline.

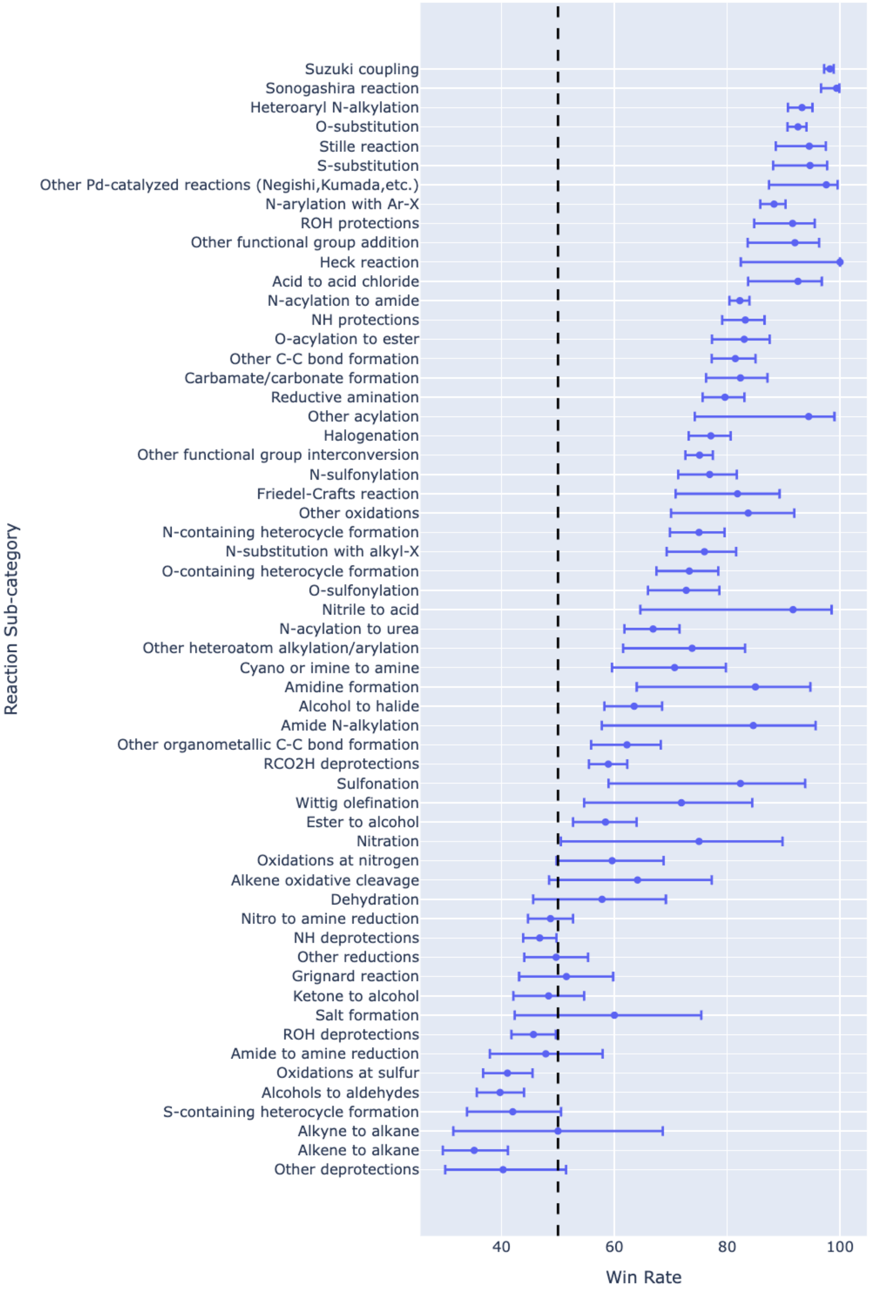

Figure S11 Win-rate analysis comparing FFN and popularity baseline.

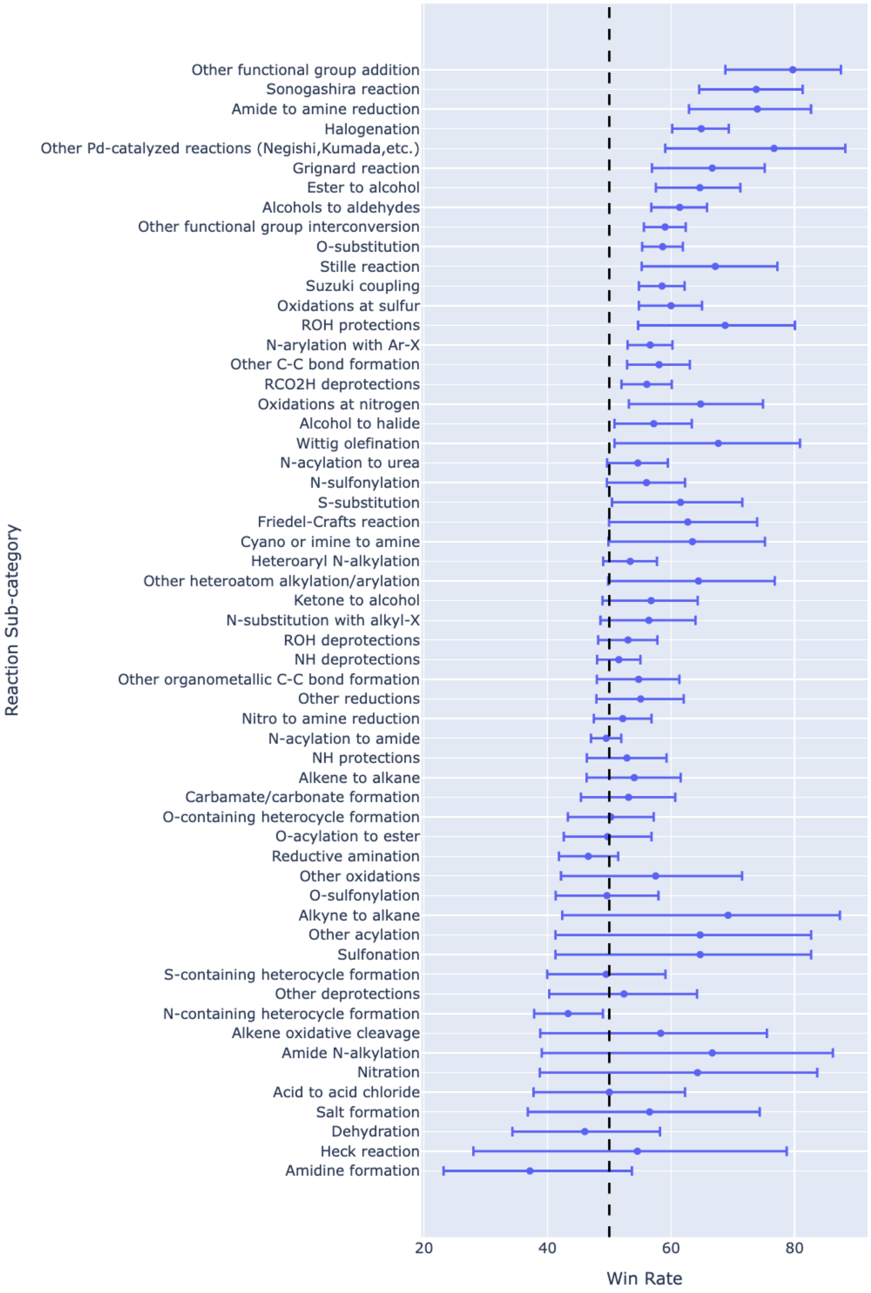

Figure S12 Win-rate analysis comparing GNN and nearest neighbor baseline.

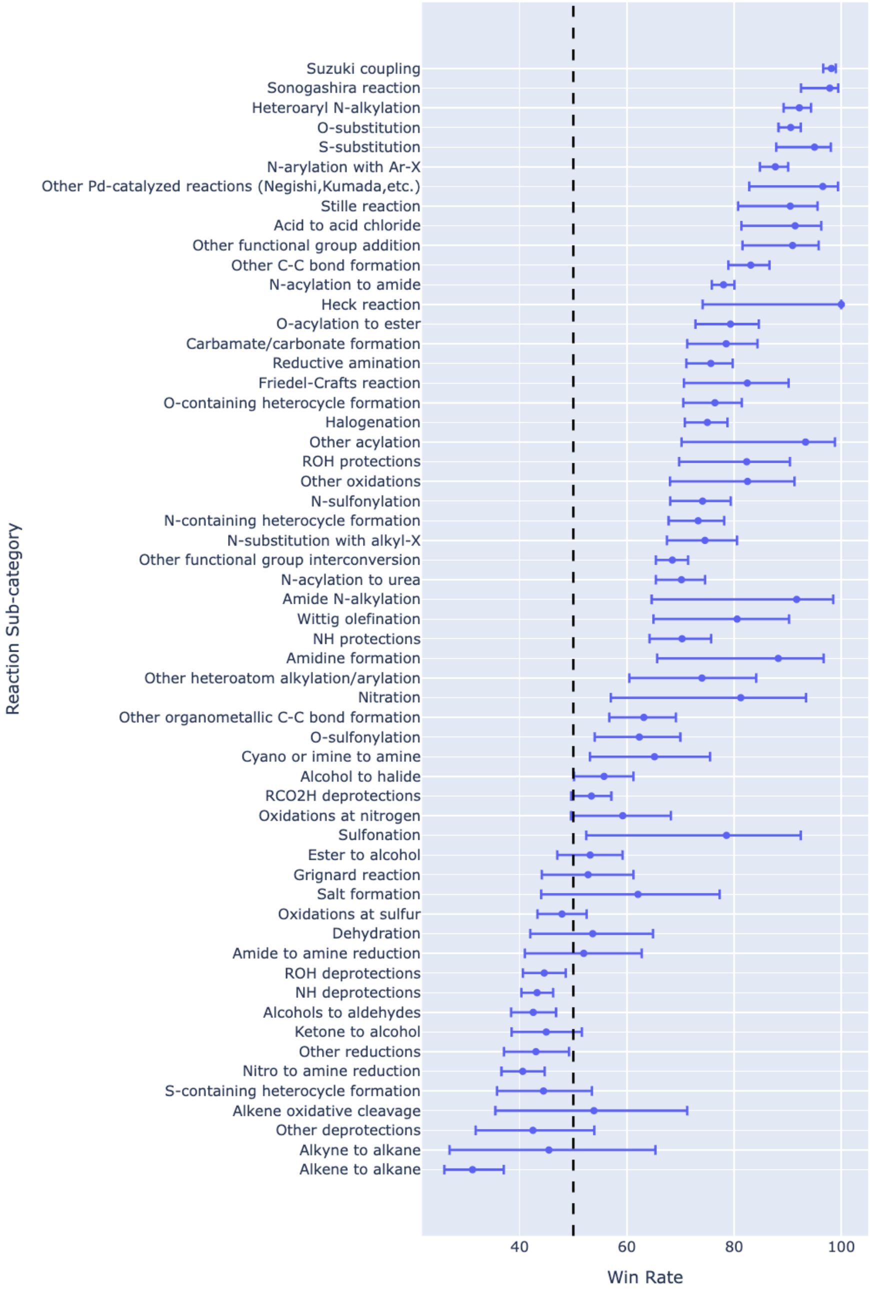

Figure S13 Win-rate analysis comparing GNN and popularity baseline.

S8 Deduplication and handling of data artifacts

A more fundamental challenge arises from parsing errors that occurred during the initial data extraction from source documents. They introduce noise that corrupts chemical records and make the deduplication procedure fail. For instance, optical character recognition mistakes can lead to misidentified reagents (e.g., “CuI” parsed as “CuL”), or incorrect numerical values (e.g., “100 °C” as “100 0C”, “– 78 °C” as “∼ 78 °C”). In some cases, entire reagents, such as “Pd(OAc)$_2$”, are missed during extraction. And work-up conditions sometimes are incorrectly lumped in as a reactive condition (Figure S14).

Such errors can cause a reaction that is a true duplicate to be incorrectly treated as unique, thereby allowing it to bypass the deduplication step. While we believe such instances are infrequent, they can artificially inflate the performance of models on those specific examples. Because these errors originate from the raw data source, they are not readily correctable within our preprocessing workflow. We acknowledge this as an inherent limitation of the dataset.

A

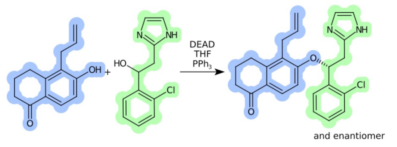

Mitsunobu aryl ether synthesis (1.7.7)

Name	Role	Formula	MW	Amount	Mass	Volume	Density	Yield
(±)-6-[1-(2-Chlorophenyl)-2-imidazolylethoxy]-5-prop-2-enyl-2,3,4-trihydronaphthal…	Product	$C_{24}H_{23}ClN_2O_2$	406.905 g/mol					
1-(2-Chlorophenyl)-2-imidazolylethan-1-ol (**compound**)	Reactant	$C_{11}H_{11}ClN_2O$	222.671 g/mol	4.9 mmol	1.1 g			
6-hydroxy-5-prop-2-enyl-2,3,4-trihydronaphthalen-1-one	Reactant	$C_{13}H_{14}O_2$	202.249 g/mol	4.45 mmol	900 mg			
triphenylphosphine	Agent	$C_{18}H_{15}P$	262.285 g/mol	4.9 mmol	1.28 g			
diethyl azodicarboxylate	Solvent	$C_6H_{10}N_2O_4$	174.155 g/mol	6.9 mmol	1.202 g	1 mL	1.202 g/mL	
tetrahydrofuran	Solvent	C_4H_8O	72.106 g/mol	308.227 mmol	22.225 g	25 mL	0.889 g/mL	
tetrahydrofuran	Solvent	C_4H_8O	72.106 g/mol	61.645 mmol	4.445 g	5 mL	0.889 g/mL	

Info

Source	US20040044057A1 [1079] EXAMPLE 59, Step 4
Document	Andrew White, Daniele Leonard, Douglas Johnson, Gordon Rewcastle, Ho Lee, James Kaltenbronn, Jared Milbank, Joseph Repine, Richard Hutchings, William Denny **5- substituted tetralones as inhibitors of ras farnesyl trransferase** U.S. Application (04-Mar-2004)
IPC Codes	A61K 31/4164, A61K 31/4178, C07D 233/60
Diseases	Atherosclerosis, Blindness, Hepatitis D, Neoplasms, Pathologic Constriction, Polycystic Kidney Diseases, Prostatic Hyperplasia, Psoriasis
Quality Flags	A No yield reported or calculable

Procedure

4. (±)-6-[1-(2-Chlorophenyl)-2-imidazolylethoxy]-5-prop-2-enyl-2,3,4-trihydronaphthalen-1-one
To a solution of 6-hydroxy-5-prop-2-enyl-2,3,4-trihydronaphthalen-1-one (Example 49, step 4) (0.9 g, 4.45 mmol) in dry tetrahydrofuran (25 mL) is added the compound from step 3 (1.1 g, 4.9 mmol) and triphenylphosphine (1.28 g, 4.9 mmol). The reaction mixture is cooled to 0° C. and treated with a solution of diethyl azodicarboxylate (1 mL, 6.9 mmol) in tetrahydrofuran (5 mL) dropwise. The reaction is warmed to room temperature and stirred overnight. The solution is concentrated and the residue is taken up in 100 mL ethyl acetate. The organic layer is washed with water (3×50 mL) and brine (2×50 mL). The solvent is removed in vacuo and 50 mL of ethyl ether is added. The precipitate is filtered and the ether is removed. More ethyl ether is added and the above procedure is repeated two more times. One hundred milliliters of ethyl acetate is added, and the solution is dried over MgSO4, filtered, and concentrated to give a light tan foam. Purification is carried out via reversed-phase HPLC (0.1% trifluoroacetic acid in acetonitrile and 0.1% aqueous trifluoroacetic acid as eluent; C-18 column) to give a white solid as the final product (Compound 59); 0.348 g (15% yield). MS-APCI: M+1=407.2.

B

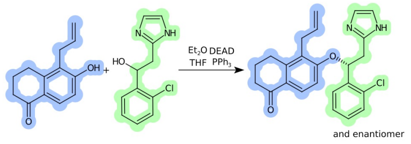

Mitsunobu aryl ether synthesis (1.7.7)

Name	Role	Formula	MW	Amount	Mass	Volume	Density	Yield
(±)-6-[l-(2-Chlorophenyl)-2-imidazolylethoxy]-5-prop-2-enyl-2,3,4-trihydronaphthale…	Product	$C_{24}H_{23}ClN_2O_2$	406.905 g/mol					
1-(2-Chlorophenyl)-2-imidazolylethan-1-ol (**compound**)	Reactant	$C_{11}H_{11}ClN_2O$	222.671 g/mol	4.9 mmol	1.1 g			
6-hydroxy-5-prop-2-enyl-2,3, 4-trihydronaphthalen- 1-one	Reactant	$C_{13}H_{14}O_2$	202.249 g/mol	4.45 mmol	900 mg			
triphenylphosphine	Agent	$C_{18}H_{15}P$	262.285 g/mol	4.9 mmol	1.28 g			
diethyl azodicarboxylate	Solvent	$C_6H_{10}N_2O_4$	174.155 g/mol	6.9 mmol	1.202 g	1 mL	1.202 g/mL	
ethyl ether	Solvent	$C_4H_{10}O$	74.122 g/mol	480.965 mmol	35.65 g	50 mL	0.713 g/mL	
tetrahydrofuran	Solvent	C_4H_8O	72.106 g/mol	308.227 mmol	22.225 g	25 mL	0.889 g/mL	
tetrahydrofuran	Solvent	C_4H_8O	72.106 g/mol	61.645 mmol	4.445 g	5 mL	0.889 g/mL	

Info

Source	WO2001079180A2 [0949]
Document	William Denny, Richard Hutchings, Douglas Johnson, James Kaltenbronn, Ho Lee, Daniele Leonard, Jared Milbank, Joseph Repine, Gordon Rewcastle, Andrew White **5-substituted Tetralones As Inhibitors Of Ras Farnesyl Transferase** WIPO PCT (25-Oct-2001)
Affiliation	Warner-Lambert
IPC Codes	C07D 233/54, C07D 233/91, C07D 401/12, C07D 521/00
Diseases	Atherosclerosis, Blindness, Hepatitis D, Neoplasms, Pathologic Constriction, Polycystic Kidney Diseases, Prostatic Hyperplasia, Psoriasis
Quality Flags	A No yield reported or calculable

Procedure

To a solution of 6-hydroxy-5-prop-2-enyl-2,3, 4-trihydronaphthalen- 1-one (Example 49, step 4) (0.9 g, 4.45 mmol) in dry tetrahydrofuran (25 mL) is added the compound from step 3 (1.1 g, 4.9 mmol) and triphenylphosphine (1.28 g, 4.9 mmol). The reaction mixture is cooled to 0°C and treated with a solution of diethyl azodicarboxylate (1 mL, 6.9 mmol) in tetrahydrofuran (5 mL) dropwise. The reaction is warmed to room temperature and stirred overnight. The solution is concentrated and the residue is taken up in 100 mL ethyl acetate. The organic layer is washed with water (3 × 50 mL) and brine (2 × 50 mL). The solvent is removed in vacuo and 50 mL of ethyl ether is added. The precipitate is filtered and the ether is removed. More ethyl ether is added and the above procedure is repeated two more times. One hundred milliliters of ethyl acetate is added, and the solution is dried over MgSO4, filtered, and concentrated to give a light tan foam. Purification is carried out via reversed-phase HPLC (0.1% trifluoroacetic acid in acetonitrile and 0.1% aqueous trifluoroacetic acid as eluent; C-18 column) to give a white solid as the final product (Compound 59); 0.348 g (15% yield). MS-APCI: M + 1 = 407.2. Analysis calculated for C24H23N2O2Cl1·1.32 C2H1O2F3· 0.75 H2O:

Figure S14 Example of deduplication failure due to parsing error. (A) A test reaction from patent US20040044057A1. (B)A train reaction record from patent WO2001079180A2, identified as the closest neighbor of A. B incorrectly lumps the work-up solvent ethyl ether.

References

[1] M. Douze, A. Guzhva, C. Deng, J. Johnson, G. Szilvasy, P.-E. Mazaré, M. Lomeli, L. Hosseini and H. Jégou, *The Faiss library*, 2025, `http://arxiv.org/abs/2401.08281`, arXiv:2401.08281 [cs].

[2] R. Liaw, E. Liang, R. Nishihara, P. Moritz, J. E. Gonzalez and I. Stoica, *Tune: A Research Platform for Distributed Model Selection and Training*, 2018, `http://arxiv.org/abs/1807.05118`, arXiv:1807.05118 [cs].
